# A mammalian, glutaminase-free asparaginase enhances venetoclax activity in preclinical AML models with chromosome 7 deletion

**DOI:** 10.3389/fonc.2025.1606239

**Published:** 2026-02-10

**Authors:** Dhabya Majid, Zhe Wang, Amanda M. Schalk, Bin Yuan, Qi Zhang Tatarata, Jessica L. Root, Araceli Isabella Garza, Mhd Yousuf Yassouf, Basant T. Gamal, Sammy Feri-Borgogno, Annie Hoai Nguyen, Marina Konopleva, Evelien Peeters, Ying Su, Patrick K Reville, Farhad Ravandi-Kashani, Arnon Lavie, Hussein A. Abbas

**Affiliations:** 1 Department of Leukemia, The University of Texas MD Anderson Cancer Center, Houston, TX, United States; 2 Department of Biochemistry and Molecular Genetics, College of Medicine, University of Illinois at Chicago, Chicago, IL, United States; 3 Enzyme by Design Inc., Chicago, IL, United States; 4 Department of Gynecologic Oncology and Reproductive Medicine, The University of Texas MD Anderson Cancer Center, Houston, TX, United States; 5 Rice University, Houston, TX, United States; 6 Department of Oncology, Montefiore Einstein, Bronx, NY, United States; 7 Department of Diagnostic Sciences, Ghent University, Ghent, Belgium; 8 Research Biologist, Biological Science Research and Development, Department of Veterans Affairs Medical Center, Chicago, IL, United States

**Keywords:** acute myeliod leukemia, AML, AML with deletion 7, combination treatment, leukemia, venetoclax, asparaginase

## Abstract

**Introduction:**

Acute myeloid leukemia (AML) remains a malignancy with poor prognosis andfrequent resistance to standard therapies, underscoring the urgent need for novel treatmentstrategies. In this preclinical study, we evaluated the anti-leukemic efficacy of EBD-300, a novelmammalian-derived asparaginase lacking glutaminase activity, in combination with Venetoclax(VEN).

**Results:**

EBD-300 monotherapy exhibited significant activity in AML cell lines harboringchromosome 7/7q deletions, which are likely dependent on extracellular asparagine due to thepresence of only a single copy of the asparagine synthetase (ASNS) gene - the enzymeresponsible for endogenous asparagine synthesis. The combination of EBD-300 with VENdecreased the IC50 values of some VEN-resistant AML cell lines and reduced the colony-formingcapacity of primary AML patient samples. In patient-derived xenograft (PDX) mouse models,EBD-300, alone or in combination with VEN, significantly reduced leukemic burden in theperipheral blood, bone marrow, and spleen, and improved overall survival in one model.

**Discussion:**

Although survival benefits were observed in some, but not all, models, suggestingpotential model-specific effects, these findings collectively support a potential therapeutic roleEBD-300 in combination with VEN in AML. While weight loss was observed, EBD-300 mayrepresent a potentially safer alternative to conventional bacterial asparaginases by mitigatingthe adverse effects typically associated with the glutaminase coactivity of the bacterialasparaginases, an observation that requires further investigation.

## Introduction

Acute myeloid leukemia (AML) is a relatively rare hematologic malignancy characterized by the uncontrolled proliferation of myeloid precursor cells in the bone marrow and peripheral blood. In the US, AML occurs at an incidence of approximately 4.11 cases per 100,000 people annually (1). The prognosis for AML varies significantly based on several factors, including age, cytogenetics, and genetic mutations. The 5-year survival rate for AML patients ranges from 35% to 40% in younger adults but drops to about 5–10% for those over 60 years of age, which constitutes >60% of patients, demonstrating a high unmet clinical need for efficacious therapeutics with high tolerability (2).

The combination of Venetoclax (VEN) and Azacitidine (AZA) is the standard of care for AML patients who are ineligible for intensive chemotherapy, achieving response rates of up to 77% in newly diagnosed cases (3). VEN, a BCL-2 inhibitor, promotes apoptosis by releasing pro-apoptotic proteins, including BAX, BAK, and BH3-only proteins, which trigger mitochondrial permeabilization and caspase activation (4). However, VEN-based therapies and conventional chemotherapy are less effective in patients with adverse cytogenetic and genetic mutations, such as chromosomal deletion 7 and TP53 mutations, which are associated with poor prognosis and therapeutic resistance (5). Therefore, there is an urgent need for more targeted therapies or novel combination approaches to improve outcomes in adverse-risk AML patients.

Asparaginase, an enzyme commonly used in the treatment of acute lymphoblastic leukemia (ALL), depletes asparagine in the blood by converting it into aspartic acid and ammonia, effectively starving leukemic cells that rely on external sources of this amino acid for survival (6). The success of asparaginase-containing regimens in the treatment of pediatric ALL, and poor outcomes with conventional cytotoxic regimens in adults, have led to trials of pediatric-inspired regimens incorporating asparaginase in the adolescent and young adult (AYA) and adult populations. Additionally, certain AML subtypes show sensitivity to asparagine depletion. Some AML subtypes with specific metabolic dependencies, such as low asparagine synthetase (ASNS) expression, may also respond to asparaginase therapy (7).

The currently available asparaginases are associated with clinical hypersensitivity in 7-30% of patients due to their bacterial origin (8). Additionally, they are linked to potentially severe non-immune reactions, such as liver toxicity and pancreatitis, due to their glutaminase co-activity (9). To address these limitations, we developed a mammalian-derived, glutaminase-free asparaginase, EBD-300, designed to minimize hypersensitivities and toxic side effects.

EBD-300 is derived from the only known mammalian asparaginase to possess the requisite kinetic properties for an anti-cancer effect, guinea pig L-ASNase (*Gp*A; Gene name ASPG; Uniprot ID H0W0T5) (). We designed EBD-300 to closely mimic the human asparaginase (*h*A) homolog in sequence – to minimize the immunogenicity -while preserving the unique kinetics of *Gp*A. This approach was necessary because *h*A has a millimolar Km for asparagine and functions as a type 1 allosteric asparaginase. Such behavior prevents it from effectively depleting asparagine at physiological concentrations, rendering it unsuitable as a clinical candidate (see asparaginase kinetics in **Table 1**). Different from the bacterial enzymes, EBD-300 has virtually no glutaminase activity. Of note, as we reported previously, so-called “low-glutaminase” bacterial asparaginases are actually impaired in their asparaginase activity due to an elevated asparagine Km (low affinity for ASN under physiological conditions). Therefore, to our knowledge, our low/no-glutaminase asparaginases (EBD-100/200/300 and an ErA variant we engineered to have low glutaminase activity (12) are the only variants with the required kinetic properties needed to achieve complete asparagine depletion *in vivo*. In this preclinical study, we investigated the anti-leukemic effects of EBD-300, both as a monotherapy and in combination with VEN, in various AML models, including cell lines, primary patient samples, and PDX models with adverse-risk mutations and cytogenetics. Our findings demonstrate the effective anti-leukemic activity of the VEN + EBD-300 combination and its reduction of AML clonogenicity, highlighting its potential as an effective treatment strategy.

**Table 1:** In vitro kinetics and in vivo pharmacokinetics for clinical bacterial and relevant mammalian L-asparaginases.

Enzyme	Asparaginase activity		Glutaminase activity				Mouse half- life
	k_cat_ (s^-1^)	k_m_ (µM)	k_cat_ (s^-1^)	K_m_ (µM)	k_obs_ at 1 mM (s^-1^)	Glutaminase/asparaginase activities (%)	t_1/2_ (hr)
*Er*A (10)	207.5 ± 3.6	47.5 ± 3.5	26.84 ± 0.26	0.36 ± 0.02	19.22	13	2.3
*Ec*A (10)	44.4 ± 0.3	15.0 ± 0.5	0.89 ± 0.01	1.38 ± 0.09	0.36	2	3.5
EBD-300	70.0 ± 0.5	41.2 ± 1.2	0.01	ND	0.00	0	20.8
*Gp*A (11)	38.6 ± 1.4	57.7 ± 6.4	0.00	ND	0.00	0	ND
*h*A (11)	14.4 ± 0.4	2,960 ± 131^a^	ND	ND	ND	ND	ND

The table summarizes the catalytic efficiency kcat and Michaelis-Menten constant Km for asparaginase and glutaminase activities of each L-asparaginase. Additionally, the *in vivo* mouse half-life is included for each enzyme.

^a^ hA shows allosteric behavior, in line with Type I asparaginases (EcA is a Type II enzyme), and for these the Km is called [S]_0.5_ or K_0.5_.

ND, Not determined.

## Results

### Development of EBD-300, a novel human-like asparaginase with no off-target glutaminase side activity

**Figure 1:**
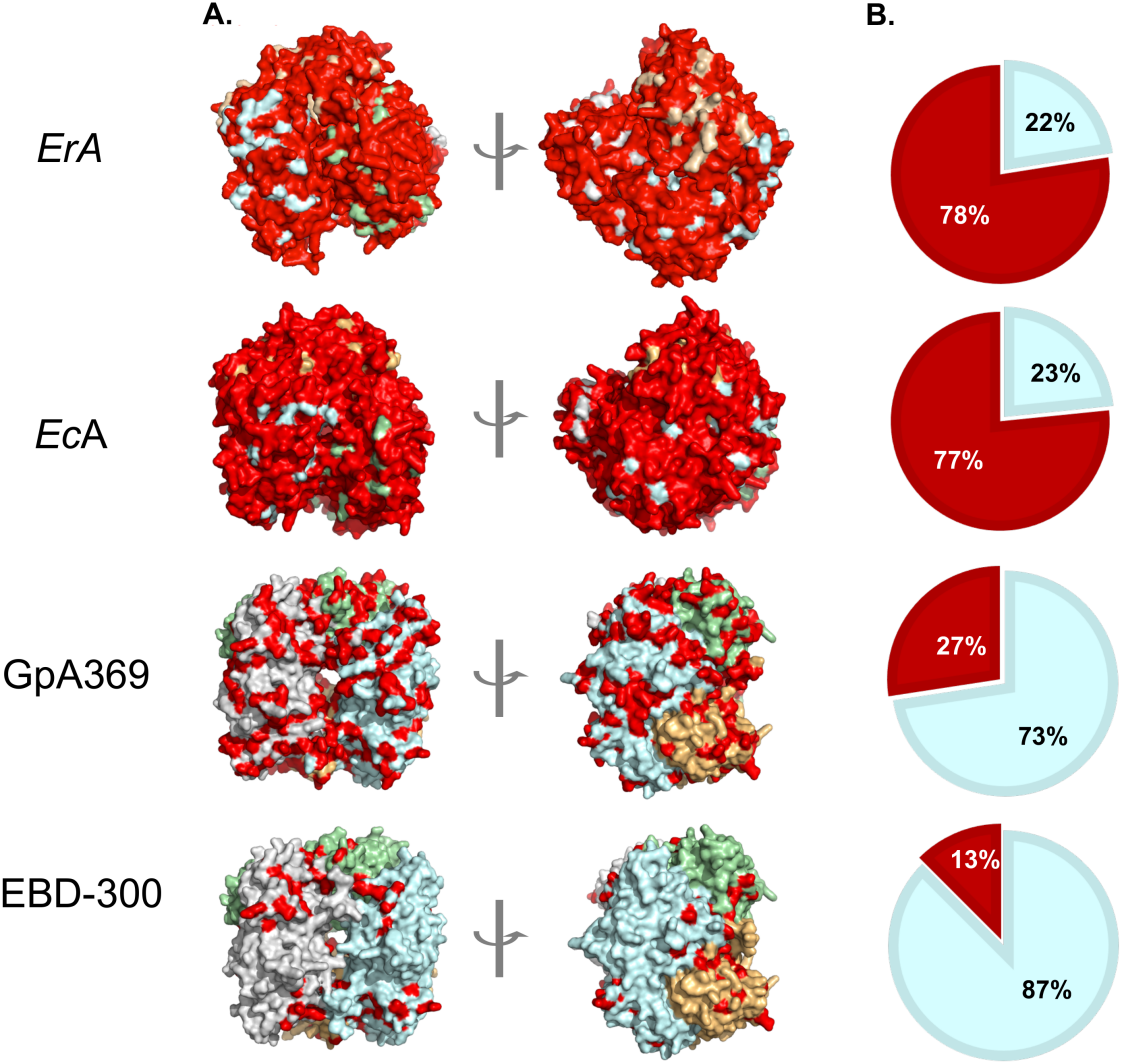
EBD-300 is highly human-like in protein sequence compared to the asparaginase domain from the guinea pig homolog truncated at residue 369 (*Gp*A369) but especially compared to the clinical bacterial asparaginases from E. coli (*Ec*A) and Erwinia (*Er*A) **(A)** surface representations of the tetramers where each protomer in the tetramer is shown in a different color (gray, green, blue or orange) and residues not identical to the human homolog are denoted in red. **(B)** Pie charts displaying % identity compared to the human enzyme where non-human is shown in red and identity to human is show in blue.

EBD-300 is a third-generation variant built on the backbone of GpA. As can be seen in the surface representations of the tetramers of the clinical bacterial asparaginases from *E. coli* (*Ec*A) and *Erwinia* (*Er*A) (**Figure 1A**) as well as the percent identity graphs (**Figure 1B**), the bacterial enzymes are highly dissimilar (<25% identity) to *h*A, which is in line with their high immunogenicity. In contrast, *Gp*A shares over 70% sequence similarity with hA, providing a strong foundation for reducing immunogenicity. To further mitigate this risk, we engineered EBD-300 by first truncating it to remove the C-terminal domain, which is absent in bacterial type II asparaginases and may carry additional enzymatic activities. The first generation, EBD-100, was generated through a structure-guided process that identified surface residues that could be mutated to the corresponding hA sequence without loss of activity, increasing the percent amino-acid identity to hA from 69.8% for GpA to 81.7% for EBD-100. Subsequently, by using a T-cell proliferation assay that used overlapping peptides containing non-human residues with human PBMCs to see which peptides were able to elicit a T-cell response, we identified several residues with the potential to induce T-cell stimulation, and these were mutated to the hA sequence, resulting in EBD-200 with 83% identity to hA. We recently reported our evaluation of EBD-200 in blood and select solid tumors that showed high anti-cancer efficacy (13). Finally, we employed a mass-spectrometry analysis of naturally processed EBD-200 to identify epitopes presented on MHC-II. While these epitopes are not guaranteed to trigger a T-cell response, elimination of these potential epitopes would abrogate the potential for a T-cell proliferation response. Therefore, many of those epitopes were likewise mutated to the hA sequence, resulting in EBD-300 that has 87.1% identity to hA. Throughout this humanization and de-immunization process we made sure to assess the functionality of the variants, with a major focus on the asparagine Km value (low Km = high affinity for substrate). To achieve complete and persistent depletion of blood asparagine, the Km value needs to be in the low micromolar range. With EBD-300 we achieved a human-like asparaginase that has a Km for asparagine of 41.2 µM, which is between that of EcA (15.0 µM) and ErA (47.5 µM) – **Table 1**. The rate of the reaction (k_cat_) is also between these two clinically used asparaginases.

EBD-300 is highly unique in that it is an asparaginase that has a low Km for, the kineticproperty required for sustained depletion of this amino acid in the blood that gives asparaginaseits anti-cancer effect in the clinic, while also demonstrating no off-target glutaminase activity,even at high concentrations of enzyme and excess glutamine substrate (**Supplementary Figure 1**; **Table 1**). This is in stark contrast to the bacterial clinical asparaginases of which*ErA* and *Ec*A demonstrate strong and moderate glutaminase activity, respectively (**Supplementary Figure 1**; **Table 1**). In addition to having the required kinetic properties to efficiently deplete blood of asparagine without impacting the all-important glutamine (thereby eliminating side effects due to perturbations in glutamine levels and high ammonia production), an additional property of EBD-300 that favors clinical use is its extended *in vivo* persistence (**Table 1**). The very short half-life of the naked bacterial asparaginases demands high doses given frequently (three times per week). The seven-fold longer half-life of EBD-300 suggests that, when administered at a similar dose, a single weekly treatment will achieve and maintain full asparagine depletion, which would improve patient experience and compliance, leading to improved outcomes. With this unique asparaginase at hand, we next aimed to explore its effectiveness in AML.

### EBD-300 enhanced the anti-leukemic activity of VEN in AML cell lines

**Figure 2:**
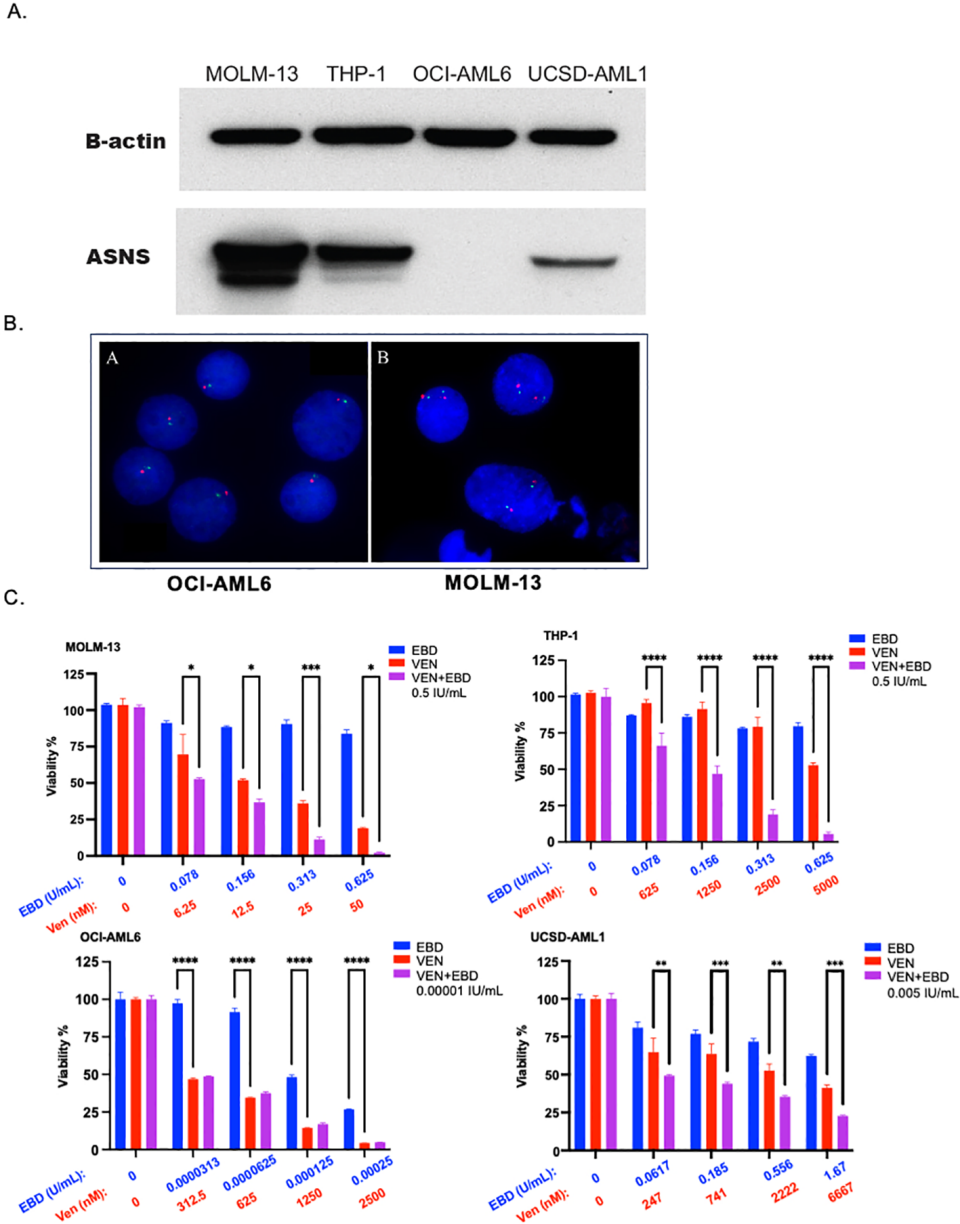
EBD-300 enhances the anti-leukemic activity of VEN in AML cell lines. **(A)** Western blot analysis (n=1) reveals lower ASNS protein levels in deletion 7 cell lines (OCI-AML6 and UCSD-AML1) compared to non-deletion 7 cell lines (MOLM-13 and THP-1). **(B)** Fluorescence in situ hybridization (FISH) analysis shows that all six OCI-AML6 nuclei have only one red (RELN) and one green (TES) fluorescence signal, indicating potential monosomy of chromosome 7 or a deletion in the 7q region. In contrast, all three MOLM-13 nuclei show two pairs of bright fluorescent spots, confirming the presence of both copies of 7q. **(C)** The cell lines (n=3, technical replicates) were treated with VEH, VEN, or VEN+EBD-300 (0.5 IU/mL) for 96 hours after that Cell titer glo (CTG) assay was used to evaluate cell death. EBD-300 enhances the anti-leukemic effect of VEN in MOLM-13, reducing the VEN IC-50 from 0.0240 µM to 0.0128 µM, and in THP-1, reducing the VEN IC-50 from 5.21 µM to 1.72 µM. OCI-AML6 showed high sensitivity to EBD-300 (IC-50 of 1.1e-4 U/mL), while UCSD-AML1 was resistant to EBD-300. However, EBD-300 still enhanced the anti-leukemic effect of VEN in UCSD-AML1, reducing the VEN IC-50 from 4.34 µM to 2.11 µM. The error bars shown represent the Standard Error of mean or (SEM). *= <0.05, **= <0.01, ***= <0.001, ****= <0.0001.

We quantified ASNS levels in two chromosome 7-deleted cell lines, OCI-AML6 and UCSD-AML1. We also measured ASNS in two cell lines without chromosome 7 deletions: MOLM-13 (VEN-sensitive) and THP-1 (VEN-resistant) (**Figure 2A**). Of note, we performed fluorescence *in situ* hybridization (FISH) on OCI-AML6 and MOLM-13 cell lines and confirmed chromosome loss in OCI-AML6, but not MOLM-13 (**Figure 2B**). The cell lines with 7/7q deletions showed lower ASNS expression compared to those without the deletions (**Figure 2A**), consistent with gene dose effect.

We next evaluated the efficacy of EBD-300 in inducing cell death in AML cell lines with and without VEN using Cell Titer Glo (CTG) assay. The anti-leukemic activity of VEN, EBD-300, and their combination (VEN+EBD-300) varied among the cell lines. In MOLM-13 cells, the combination of VEN and EBD-300 (IC50 = 6.84 nM, 95% CI: 6.29 - 7.64, n=3, **Figure 2C**) enhanced sensitivity compared to VEN alone (IC50 = 8.66 nM, 95% CI: 5.83 - 12.76, n=3, **Figure 2C**). A strong synergistic effect was observed in THP-1 cells, a VEN-resistant model (14). The VEN IC50 was markedly reduced from 2268.8 nM (95% CI: 1528.3 - 3120.8, n=3, **Figure 2C**) to 953.9 nM (95% CI: 642.6 - 1416.1, n=3, **Figure 2C**) when combined with EBD-300. Conversely, OCI-AML6 cells were highly sensitive to EBD-300 monotherapy (IC50 = 0.000101 IU/ml, 95% CI: 0.000084 - 0.000117, n=3, **Figure 2C**). Given the profound sensitivity to EBD-300 monotherapy, no appreciable reduction in the VEN IC50 was observed with the combination therapy; the IC50 remained at 312.5 nM (95% CI: 291.6 - 351.6, n=4, **Figure 2C**), comparable to VEN alone (291.6 nM, 95% CI: 291.6 - 321.3, n=4, **Figure 2C**), suggesting that EBD-300 use precluded any further sensitization to VEN. Lastly, UCSD-AML1 did not exhibit sensitivity to EBD-300 monotherapy with an IC50 of 2.43 IU/mL (95% CI: 0.29 - 20.71, n = 3, **Figure 2C**); however, its combination with VEN reduced the IC50 of VEN from 2,570.3 nM (95% CI: 1,912.4 - 3,454.2, n = 3, **Figure 2C**) to 1,055.7 nM (95% CI: 904.6 - 1,231.9, n = 4, **Figure 2C**), suggesting an additive effect between VEN and EBD-300.

### EBD-300 alone or in combination with VEN, can effectively reduce colony forming potential of adverse-AML primary samples

**Figure 3:**
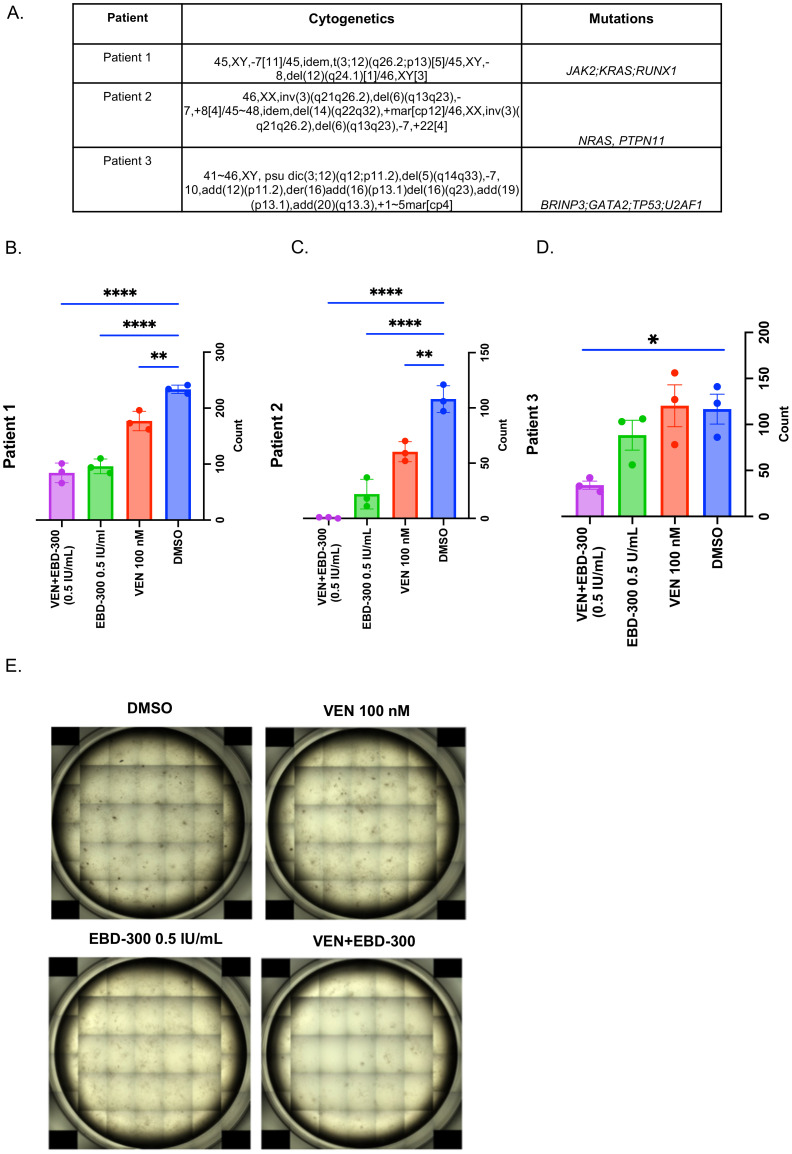
EBD-300 and VEN+EBD-300 reduce CFU counts in CD34+ primary patient samples. **(A)**Cytogenetic profiles and mutations of the primary patient samples utilized in the methylcellulosecolony-forming unit (CFU) assay are detailed. Fresh primary patient cells (n=3, technical replicates) were enriched for CD34+ and seeded in a 12-well format, with four treatment conditions, each performed in triplicate. Complete data showing three biological replicates are presented in **Supplementary Figures 3**–**5**. **(B)** In Patient 1, VEN significantly reduced CFU counts by a mean of 56.7 colonies (95% CI 18.16-95.18) compared to VEH (P=0.0059). EBD-300 also led to a significant reduction of 137.7 colonies (95% CI 99.2-176.2) compared to VEH (P<0.0001), with the VEN+EBD-300 combination further decreasing CFU counts by 149.3 colonies (95% CI 110.8-187.8) compared to VEH (P<0.0001). **(C)** In Patient 2, both VEN and EBD-300 significantly reduced CFU counts by 47.67 and 86 counts respectively in comparison to VEH (P=0.0019) and (P<0.0001). The combination of VEN+EBD-300 also significantly reduced CFU counts by 107.3 colonies (95% CI 80.8-133.8) when compared to VEH (P<0.0001). These findings support the potential efficacy of EBD-300 and VEN+EBD-300 in reducing AML stemness in primary patient samples. **(D)** In Patient 3, VEN did not significantly decrease CFU counts compared to VEH. Similarly, EBD-300 alone did not show a significant reduction. However, the VEN+EBD-300 combination resulted in a significant decrease of 82.7 colonies compared to VEH (P=0.0292). The error bars shown represent the standard error of mean (SEM). * = <0.05, **= <0.01, ****= <0.0001.

To assess the ability of EBD-300 to reduce clonogenic potential of AML primary patient cells, we performed methylcellulose-based Colony Forming Unit (CFU) assays on CD34+ enriched primary AML samples from three patients with deletion 7: Patient 1: (JAK2, KRAS, RUNX1; -7); Patient 2: (NRAS, PTPN11; -7), and Patient 3: (BRINP3L, GATA2, TP53, U2AF1; -7) (**Figure 3A**). We hypothesized that deletion 7 (where *ASNS* gene is located) would confer increased susceptibility of AML cells to EBD-300 given their dependency on exogeneous asparagine. Primary cells were cultured in methylcellulose and with DMSO, VEN (100 nM), EBD-300 (0.5 IU/mL), and a combination of VEN+EBD-300 for 14 days. For Patient 1, there was a significant reduction in CFUs with a decrease of 24.3% in the VEN group (p = 0.0066), 58.9% in the EBD-300 group (p < 0.0001), and 63.9% in the VEN+EBD-300 group (p = 0.0002) compared to the DMSO control, (**Figure 3B**; **Supplementary Figure 2**). In Patient 2, no colonies were observed, only clusters. Yet, a significant reduction in number of clusters was noted: 44.1% in the VEN group (p = 0.0055), 79.6% in the EBD-300 group (p = 0.0012), and 99.4% in the VEN+EBD-300 group (p = 0.0001) (**Figure 3C**; **Supplementary Figure 3**). Patient 3 exhibited a 24.3% reduction in CFUs with EBD-300 treatment and a 70.8% reduction with the VEN+EBD-300 combination compared to the DMSO control, while VEN alone did not result in a reduction in CFUs relative to the DMSO control (**Figure 3D**; **Supplementary Figure 4**). These findings suggest that EBD-300, either as monotherapy or in combination with VEN, can effectively reduce colony forming potential of primary leukemic cells an average of 54.4% in EBD-300 and 78.0% in VEN+EBD-300 groups. Of note, all patients had cytogenetic confirmation of deletion 7 suggesting heightened sensitivity of these cells to asparagine depletion.

### EBD-300 monotherapy inhibits leukemic burden in a del7q AML PDX model

**Figure 4:**
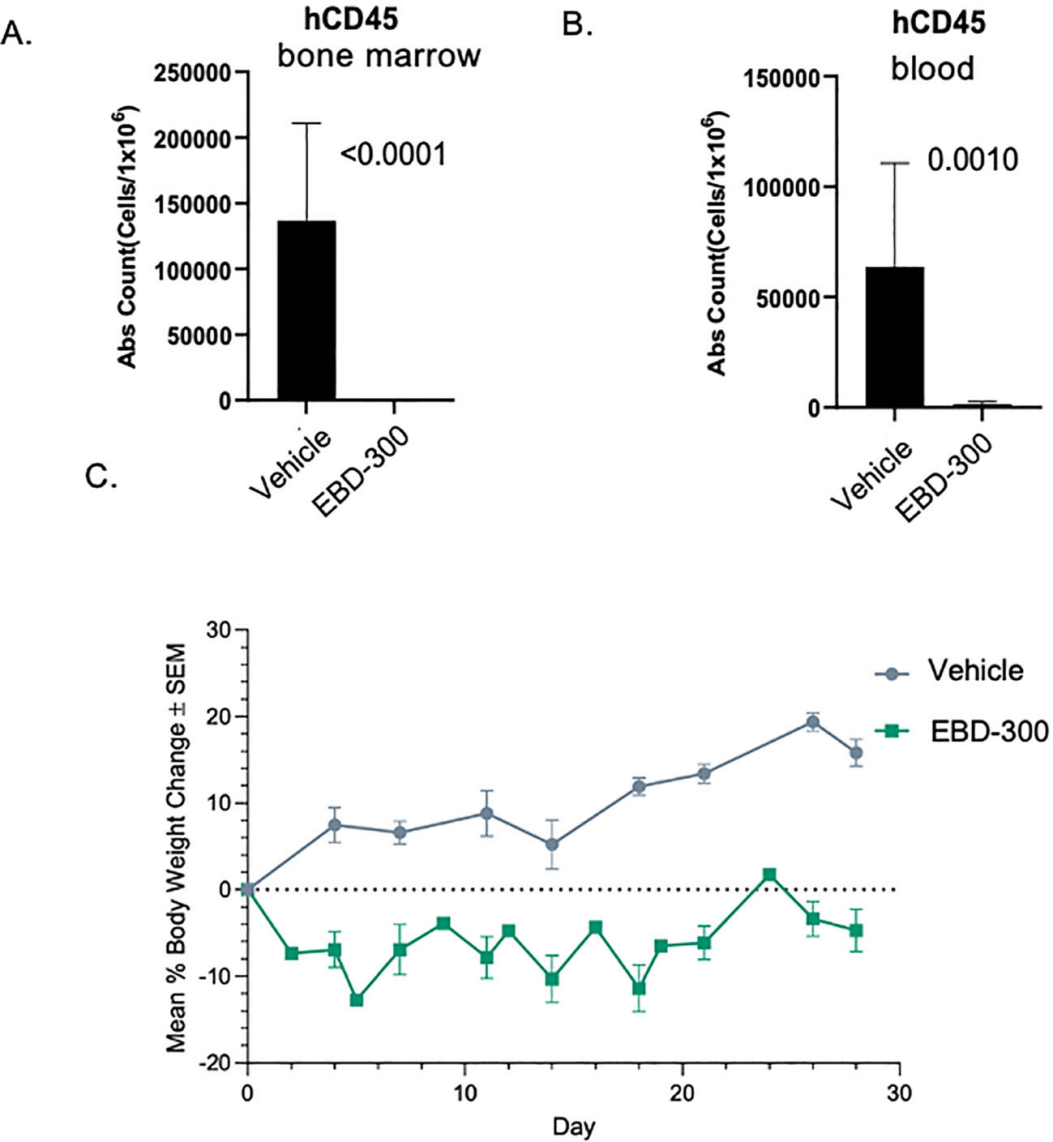
EBD-300 monotherapy suppressed leukemic growth in a del7q AML model. Human AML cells (Champions Oncology model CTG-2456) with cytogenetics 46, XX, del(7)(q22q36) [15] were implanted in NOG mice (n=10) and after confirming engraftment were randomly assigned to a vehicle control group (n=10) and an EBD-300 group (n=10), which was dosed at 750 IU/kg Mondays and Wednesdays and 1,500 IU/kg on Fridays for a total study duration of 28 days. On Day 28 mice were sacrificed, and bone marrow **(A)** and blood **(B)** were analyzed for the presence of hCD45. The animals experienced an initial weight loss 10-15% which then stabilized for the duration of the study **(C)**. Statistical significance was determined using Student t-test (Prism 9.1.2). The error bars shown represent the standard error of mean (SEM).

To further test the correlation between loss of one *ASNS* allele on chromosome 7 and sensitivity to asparaginase, we first assessed the sensitivity of a PDX model (CTG-2456) with cytogenetics 46, XX, del(7)(q22q36). Sub-lethally irradiated female NOG mice were inoculated with 2x10^6^ CTG-2456 human AML cells. Mice were monitored post-AML inoculation to assess human AML engraftment using human-CD45 (hCD45), mouseCD45 (mCD45), human-CD33, human-CD3 antibodies and BD TruCountTM beads. When individual animals had ≥ 20% live human-CD45 cells in the bone marrow, they were randomized to vehicle control and EBD-300 (n = 10/group) and were treated intravenously for 28 days. EBD-300 was given at a dose of 750 IU/kg on Mondays and Wednesdays, and 1,500 IU/kg on Fridays to account for the extended interval of the weekend. Terminal blood and bone marrow samples were collected for flow cytometry on Day 28. An 8-color panel, which targeted human CD45+ leukocytes, AML monoblasts, and Leukemic Stem Blasts, was utilized to evaluate the anti-tumor activity of EBD-300. At Day 28, the human AML cells were mostly eradicated from both the blood and bone marrow (**Figure 4A**, p <0.0001 and **Figure 4B**, p<0.001). Mice treated with EBD-300 experienced moderate weight loss that allowed for uninterrupted treatments for the entire 28 days of the study (**Figure 4C**). This strong response to EBD-300 encouraged us to expand the studies to additional AML PDX models and to combination with VEN.

### EBD-300 and VEN+EBD-300 inhibited leukemic burden in AML in preclinical models

**Figure 5:**
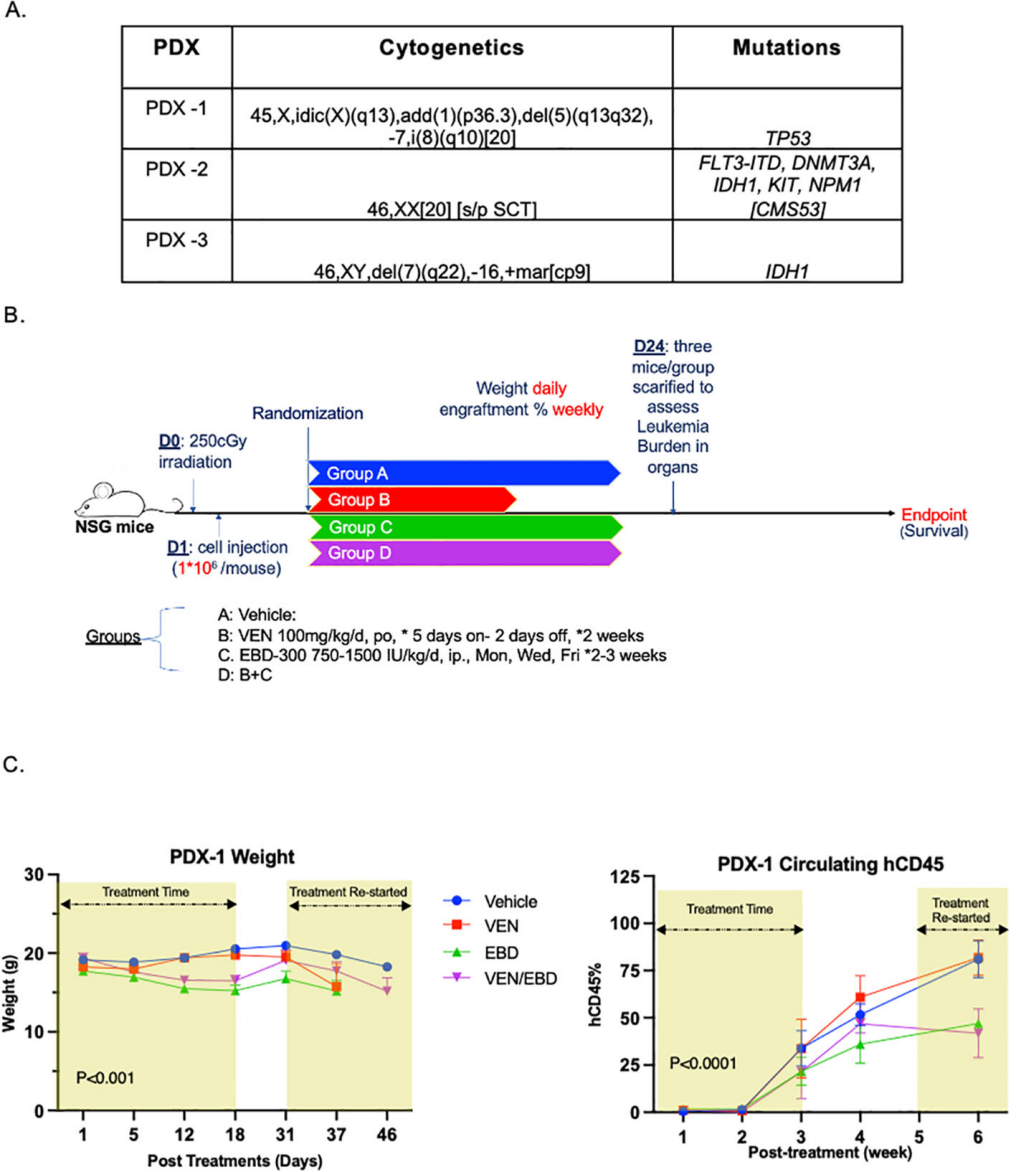
VEN+EBD-300 suppresses leukemic growth *in vivo* in adverse-risk AML models. **(A)** Cytogenetic profiles and mutations of the AML PDX models are listed. **(B)** Schematic representation of the treatment plan for the PDX models. **(C)** In the PDX-1 model, mice (n= 32) treated with EBD-300 and VEN+EBD-300 showed greater weight loss compared to those in the VEN and VEH groups (P<0.001). Additionally, the VEN and VEN+EBD-300 groups exhibited a lower percentage of circulating hCD45+ cells compared to the VEH and EBD-300 groups. The error bars shown represent the standard error of mean (SEM). .

Following the demonstration of the *invitro* efficacy of VEN+EBD combination and *in vivo* EBD-300 single agent efficacy, we further tested the preclinical efficacy of VEN, EBD-300 and the combination in three PDX models with different mutations and cytogenetic profiles, described in **Figure 5A**. Briefly, following sublethal irradiation, immunodeficient NSG mice were injected with 1x10^6^ PDX cells. Engraftment was monitored weekly via measuring the hCD45 levels via flow cytometry in peripheral blood; and upon confirmation of engraftment, the mice were randomized into four groups for treatments: Vehicle control (VEH), Venetoclax (VEN), EBD and the combination of Venetoclax and EBD (VEN+EBD-300) as outlined in **Figure 5B**.

For PDX-1 (*TP53*-mutated; deletion 7; adverse risk), EBD-300 was administered at a dose of 750 IU/kg intraperitoneally twice a week for three weeks, while other treatments followed the dosages listed in **Figure 5B**. The average circulating hCD45 percentage was lower in the EBD-300 and VEN+EBD-300 groups, at 21.64% and 21.45% respectively, compared to 33.7% in the VEH group and 33.8% in the VEN group at week3 (**Figure 5C**). Notably, circulating hCD45% began to rise again by week 4 after the termination of treatment. Therefore, we resumed the EBD-300 treatment at week 5 to evaluate whether it could once again inhibit the progression of AML. After resuming treatment, circulating hCD45% further decreased to 47.0% in the EBD-300 group and 41.8% in the VEN+EBD-300 group by week 6, compared to 81.0% and 81.8% in the VEH and VEN groups (VEN+EBD-300 vs VEH, P<0.029, VEN+EBD-300 vs VEN, P<0.005, **Figure 5C**). Mice receiving EBD-300 (Groups EBD-300 and VEN+EBD) exhibited a 22.5% decrease in body weight by day 18 compared to the VEH group (**Figure 5C**, p <0.001). This is consistent with the asparaginase-based weight loss seen in murine studies (12). Moreover, the weight of mice in those groups recovered after halting treatment from Day 18 to Day 31, with an increase of 7.19% and 13.6% in the EBD-300 and VEN+EBD-300 groups, respectively. On Day 24, three mice from each group were sacrificed to assess the leukemia burden in the bone marrow, spleen, and liver. There were no significant differences in hCD45% across the VEN, EBD, and VEN+EBD-300 groups (**Supplementary Figure 5**). Nevertheless, the VEN+EBD-300 treatment group exhibited a prolonged median survival of 60 days, compared to 52 days, 54 days, and 49 days observed in the VEH, VEN, and EBD-300 groups, but this was not statistically significant (P = 0.364) (**Supplementary Figure 5**). This suggests that while the burden of the disease in the organs and the survival outcomes may not have been significantly different likely due to the aggressive nature of the disease but EBD-300 and VEN+EBD-300 still reduced the circulating hCD45%.

**Figure 6:**
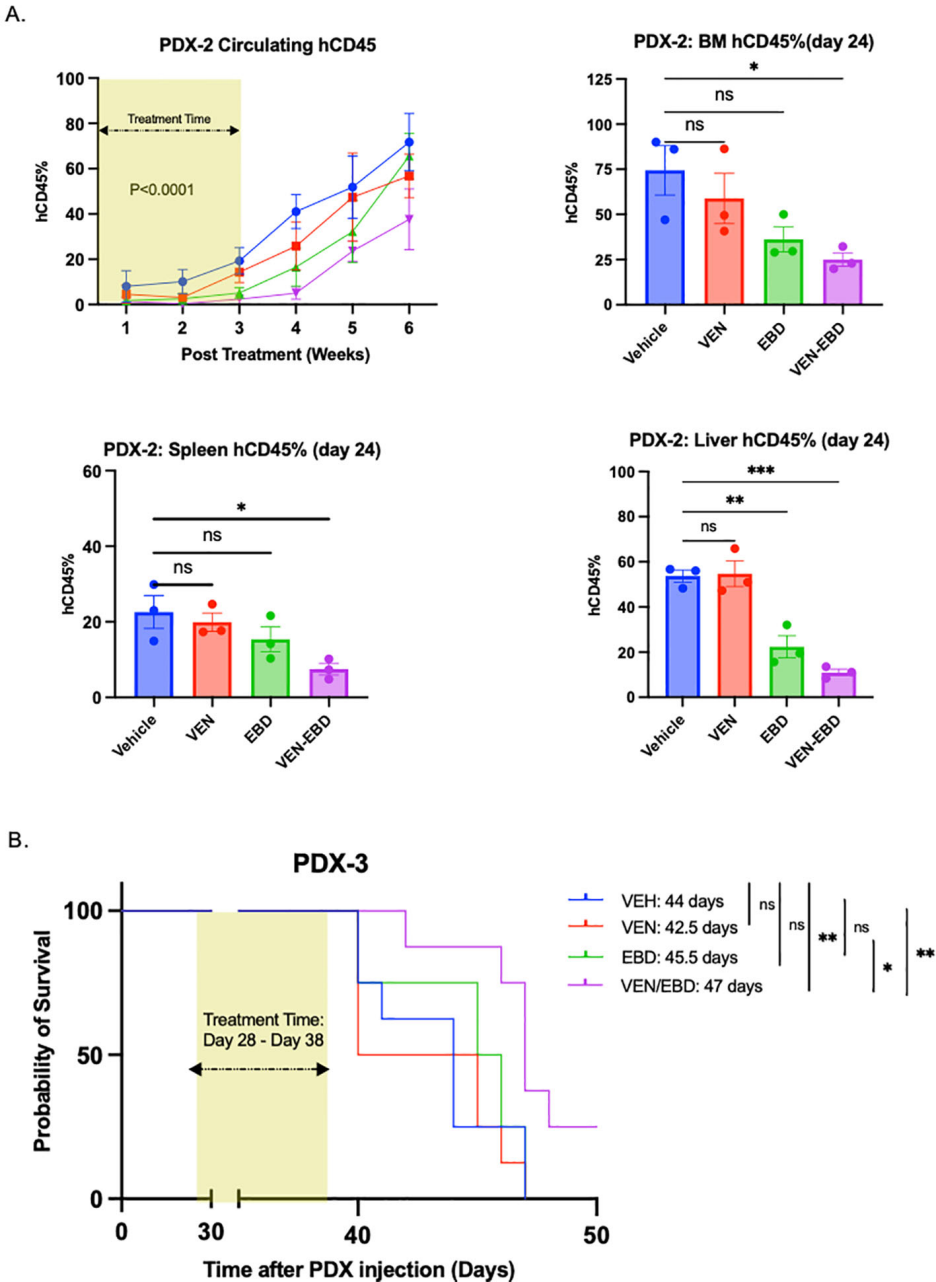
VEN+EBD-300 suppresses leukemic growth *in vivo* in adverse-risk AML models. **(A)** In the PDX-2 model (n=32)l, the EBD-300 and VEN+EBD-300 groups had significantly lower circulating hCD45+ cell percentages compared to the VEH group (P<0.0001). VEN+EBD-300 treatment also significantly reduced leukemia burden in the bone marrow compared to VEH (P=0.0266). In the spleen, both EBD-300 (P=0.0027) and VEN+EBD-300 (P=0.0003) treatments significantly reduced leukemia burden compared to VEH. The VEN+EBD-300 combination showed a significantly lower leukemia burden in the spleen compared to VEH (P=0.0209). **(B)** VEN+EBD-300 treatment increased overall survival in the PDX-3 model (n=32) compared to VEH (P=0.0159). The error bars shown represent the standard error of mean (SEM).

We hypothesized that a higher dose of EBD-300 may better control the disease. We thus testedPDX-2 (IDH1,NPM1;46,XX [20]), where we increased EBD-300 dosage to 1,500 IU/kg three times a weekfor three weeks in both the EBD-300 and VEN+EBD-300 groups. By Day 18, mice in the EBD-300 andVEN+EBD-300 groups exhibited a weight reduction of 21.2% and 13.4%, respectively, from baseline (Day 1) (P<0.0001) (**Supplementary Figure 6A**). Additionally, a significant reduction in circulating hCD45% was observed in the VEN and VEN+EBD-300 groups compared to the VEH group (p < 0.0001) (**Figure 6A**). On Day 24, three mice from each group were sacrificed, and hCD45% was assessed in the spleen, bone marrow, and liver (**Figure 6A**). There was a significant reduction in leukemia burden in the bone marrow where hCD45% levels were significantly lower in the VEN+EBD-300 group compared to the VEH group, specifically, 25.0% and 74.4%, respectively (P<0.0256) (**Figure 6A**). The VEN group had 58.9% and the EBD-300 group had 36.2% in bone marrow. Similarly, a significant reduction in leukemia burden was observed in the spleen in the VEN+EBD-300 group compared to the VEH group, (7.5% and 22.6%, respectively (P = 0.0302) (**Figure 6A**) The VEN and EBD-300 groups had a spleen leukemia burden of 19.9% and 15.4%, respectively. Furthermore, a significant decrease in leukemia burden was observed in the liver in both the EBD and VEN+EBD-300 groups compared to the VEH group, with hCD45% levels of 22.4% (P = 0.0051) and 11.0% (P = 0.0002), respectively, compared to 53.7% in the VEH group. The VEN group had an hCD45% of 54.7% (**Figure 6A**). Survival analysis for the remaining 5 mice in each group indicated no significant differences in the probability of survival between the VEN, EBD-300, VEN+EBD-300, and VEH groups (p = 0.266) (**Supplementary Figure 6A**).

Subsequently, we evaluated the drug efficacy in another PDX model, PDX-3, a deletion 7q modelwith *IDH1* mutation. EBD-300 dosage of 1,500 IU/kg was planned to be administeredthree times a week for three weeks. However, due to the aggressive nature of this leukemia, some mice started dying during the treatment, and thus the treatment was terminated at the end of the second week, and mice were subsequently assessed for survival by Kaplan-Meier analysis. Day 11 post-treatment, the EBD-300 and VEN+EBD-300 groups experienced weight losses of 15.8% and 15.6%, respectively, compared to the baseline (p=0.0203 **Supplementary Figure 6B**). Moreover, reductions in circulating hCD45% were observed in EBD-300 and VEN+EBD-300 groups with an average circulating hCD45% of 48.0% and 42.8%, respectively, in comparison to 71.8% in VEH and 82.1% in VEN groups by week 2. Survival analysis for PDX-3 demonstrated a statistically significant but modest increase in median survival to 47 days in VEN+EBD in comparison to 45.5 days, 42.5 days, and 44 days in EBD-300 (HR: 0.9362,95% CI: 0.3395 to 2.582, p=0.0233), VEH (HR: 0.9681,95% CI: 0.3510 to 2.670, p=0.0173) and VEN (HR: 0.9043,95% CI: 0.3279 to 2.494, p=0.0054), respectively (**Figure 6B**), highlighting the efficacy of VEN+EBD-300 in prolonging survival even in aggressive, high risk PDX AML models.

### Moderate weight loss but no sign of liver toxicity after repeated administration of EBD-300 in mice

**Figure 7:**
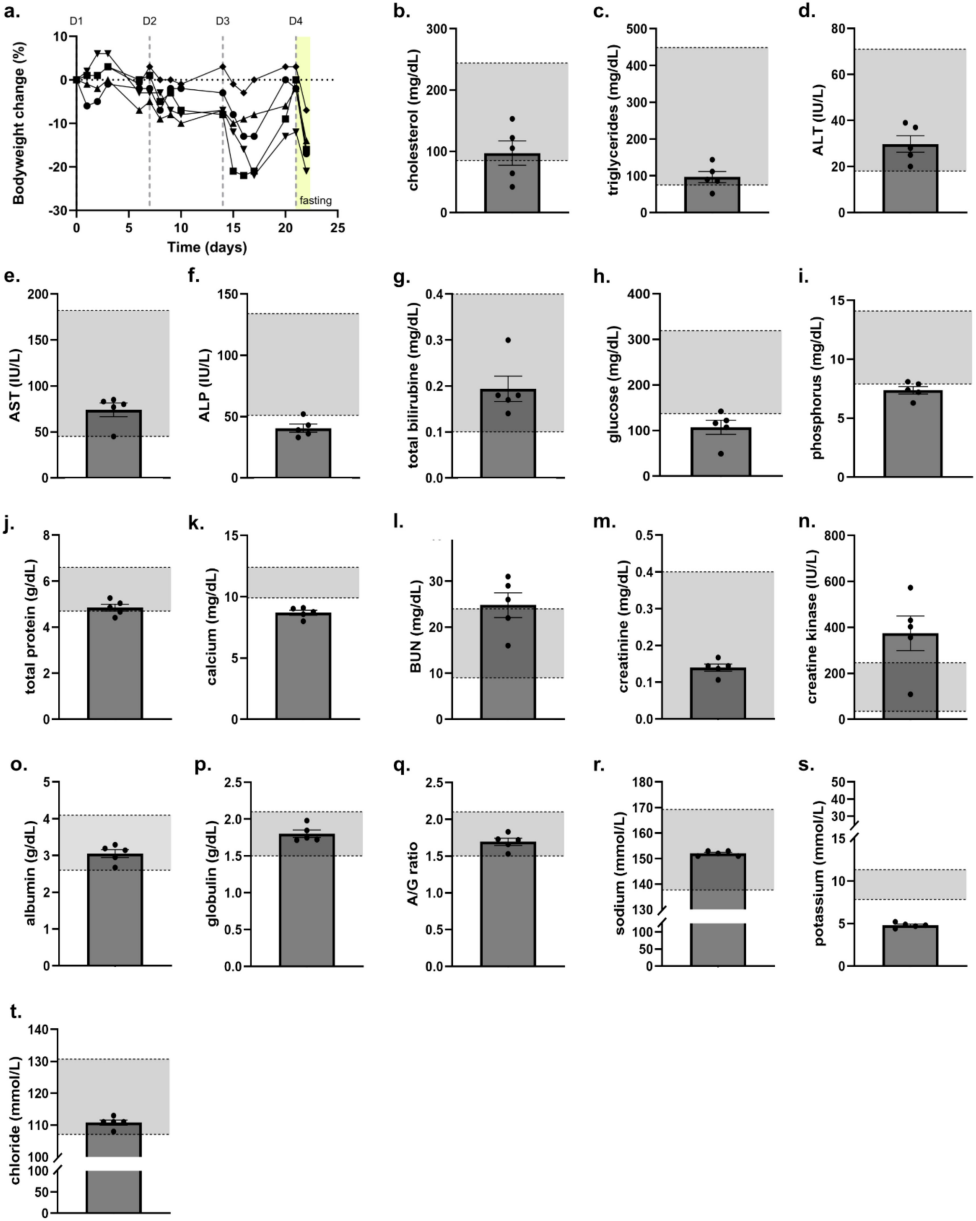
Repeat-dose toxicity study with EBD-300 shows moderate weight loss without liver toxicity. Female CD-1 mice (n=5) were administered EBD-300 i.p. at a dose of 2,000 IU/kg once/week. After the 4^th^ administration, the mice were fasted for 18 h before being euthanized and blood collected for clinical chemistry analysis. **(A)** Percent body weight changes for each mouse during the study. The larger weight loss at around the third administration can be partially due to the delivery of new mice for another study. The fasting period is highlighted in yellow. **(B–T)** Clinical chemistry results. Shown are the individual values for each animal. Reference ranges (highlighted in gray) are 95% confidence intervals of female CD-1 mice determined by Charles River and published in their CD-1 datasheet.

As noted in myriad studies, asparaginase often causes severe weight loss in mice. A recent article correlated that weight loss to an in increase in growth differentiation factor 15 (GDF15), a hormone that impacts hunger (15). Hence, the weight loss observed in mice seems to be due to lack of appetite because of increased GDF-15 levels. Of note, even mice that have lost ~20% bodyweight under EBD-300 treatment were observed to be highly active and well groomed. Given that liver injury is a frequent toxicity of clinical asparaginases, we conducted a repeat dose toxicity study in CD-1 mice in which EBD-300 was administered once/week for 4 weeks at a dose of 2,000 IU/kg. At this dose, the nadir average asparaginase activity after 1 week was determined to be ~0.5 IU/ml, which is 5-fold higher than the accepted asparaginase activity of 0.1 IU/ml required for asparagine depletion. Clinical chemistry analysis revealed normal liver values, suggesting that in mice at this dose EBD-300 does not cause liver toxicity (**Figure 7**).

## Discussion

Asparaginases, such as pegylated-crisantaspase (PegC), have shown efficacy in combination with VEN in AML pre-clinical models (16). There is a need to identify the next generation asparaginase that has the required low Km for asparagine imperative for clinical efficacy but without the excessive perturbation caused by co-activity from glutaminase. EBD-300 was designed to overcome the toxicity hurdles of current versions that prevent their widespread use in indications comprising mainly adults for which there is preclinical evidence that specific tumors will likely be sensitive to exploitation of amino acid starvation as a metabolic weakness. Current FDA approved asparaginases are associated with toxicities not only arising from their bacterial origins, but also due to their concomitant off-target glutaminase activity whereby in addition to hydrolyzing asparagine, they also hydrolyze glutamine, which is the most abundant amino acid in the blood and contributes to a myriad of essential biologic functions (12). Perturbation of glutamine homeostasis causes a wide range of side effects such as, for pegaspargase, pancreatitis (grade >2) 10-12%, thrombosis or bleeding (grade >2) 7-10% (17). Additionally, the generation of additional ammonia, especially by asparaginase Erwinia chrysanthemi that has very high glutaminase activity, can result in neurotoxicity (18).

This study is the first to demonstrate that an asparaginase lacking glutaminase activity, such as EBD-300, can also be effective as single agent or in combination with VEN in AML. EBD-300 is highly unique in that it has the combined sought-after properties of low asparagine Km enabling sustained asparagine depletion *in vivo* while being super specific for asparagine without any off-target glutaminase activity. It has further advantages over any bacterially derived asparaginases in that it is highly human-like while retaining the asparaginase activity kinetic profiles that enable clinical efficacy of the bacterial FDA-approved asparaginases.

We explored the utility of this drug in AML with a focus on deletion 7, *where ASNS* is located, and is thus thought to have higher dependency on exogenous asparagine. Specifically, ASNS catalyzes the ATP-dependent conversion of aspartic acid to asparagine. Elevated ASNS expression reduces cellular reliance on external asparagine by enabling *de novo* synthesis, conferring resistance to asparagine depletion. Conversely, ASNS downregulation, such as through promoter methylation observed in certain cancers like ALL increases the sensitivity of cells to asparagine depletion, making them more vulnerable to therapies targeting asparagine metabolism (19).

Metabolic rewiring in AML is complex, as AML cells can often adapt to metabolism inhibitors, by developing resistance mechanisms through interaction with stromal cells in the bone marrow microenvironment (20). Also, AML cells could adapt their metabolism based on the available nutrients or oxygen levels. For example, AML cells can adapt to low glucose levels by increasing their expression for GLUT5 which transports fructose (21). However, our study demonstrates that metabolic rewiring in AML is possible through amino acid depletion, which could also increase AML cells susceptibilities and sensitivities to other drugs such as VEN. Specifically, we assessed EBD-300’s efficacy, both alone and in combination with VEN, across various AML cell lines. Notably, AML cell lines with chromosome 7 deletion and low ASNS expression, like OCI-AML6, were especially sensitive to EBD-300, underscoring a potential connection between ASNS levels and treatment response. Additionally, the VEN+EBD-300 combination outperformed VEN alone in both VEN-sensitive (MOLM-13) and VEN-resistant (THP-1) cell lines, indicating that EBD-300’s depletion of asparagine enhances AML cells’ susceptibility to VEN (**Figure 2**). In primary AML samples with chromosome 7 deletion, EBD-300, alone and with VEN, reduced colony-forming potential, as seen in patient samples (**Figure 3**). This combination also lowered circulating hCD45% in PDX-1 (deletion 7 with TP53 mutation) and PDX-3 (deletion 7q) and significantly extended overall survival in the VEN+EBD-300-treated group. Our findings suggest that EBD-300, particularly with VEN, might be especially beneficial in AML cases with chromosome 7 deletion. Also, testing this combination in MDS with chromosome 7 deletion where disease burden is lower could be valuable, given that this subtype of MDS has a high risk of progressing to AML, despite being less proliferative than AML (22). Since VEN+EBD-300 reduced clonogenic growth in AML, it may have the potential to inhibit the growth and progression of clonal MDS to AML.

Future research is needed to address biomarkers that could identify patients most likely to respond to EBD-300 and VEN+EBD-300 beyond the deletion in chromosome 7, as not all patients with chromosome 7 have low ASNS levels. For instance, although AML with chromosome 7 deletion showed high sensitivity to EBD-300 in both *in vitro* and *in vivo* settings, PDX-2, which lacks chromosomal abnormalities, also responded well to EBD-300 and VEN+EBD-300, suggesting that other epigenetic regulators may influence AML cells’ susceptibility to amino acid depletion. Further studies to understand these epigenetic factors and their relationship to amino acid targeting are essential, as well as to more deeply characterize the synergistic effects of EBD-300 and VEN are warranted.

As with other targeted therapies, a potential limitation is the risk of resistance development to EBD-300 over time. Our study was not designed to evaluate the long-term resistance mechanisms of EBD-300 as a monotherapy or in combination with other drugs. Thus, future studies are required to determine whether AML could develop resistance mechanisms and to explore strategies to overcome such resistance. Another limitation of this study is that the number of the preclinical models do not fully demonstrate the heterogeneity and broad subtypes of AML patients. Hence, further studies are required to evaluate the efficacy of EBD-300 and its combination with Venetoclax in a wider range of AML subtypes in order to better identify the mechanism of action and the patient populations most likely to benefit from this therapy. Another limitation is that although, our findings show anti-leukemic activity against AML cell lines, primary patient samples and PDX models, these studies remain preclinical and the biological differences between mouse models and human AML patients limit the translation of our results. Thus, our work provides a rationale for further development and testing of EBD-300 with and without venetoclax therapy in order to establish its efficacy and safety in AML patients. Lastly, more studies are required to specify the toxicity and immunogenicity profiling versus the bacterial comparators.

## Materials and methods

### Primary AML specimens

Primary human leukemia cells were acquired via an IRB protocol named “Multimodal analysis to dissect the tumor intrinsic and microenvironmental characteristics of myeloid neoplasms” with protocol number 2022–0576 at MD Anderson Cancer Center. Mononuclear cells were purified by Ficoll-Hypaque density centrifugation and washed with complete RPMI media containing 20% FBS. CD34^+^ AML hematopoietic stem/progenitor cells (HSPCs) were purified by immunomagnetic beads conjugated with anti-CD34 antibody (StemCell Technologies) prior to utilization in colony-forming unit (CFU) assays.

### Cell lines and culturing

The human AML cell lines MOLM-13 and THP-1 were generously provided by Dr. Marina Konopleva from The University of Texas MD Anderson Cancer Center. OCI-AML6 and UCSD-AML1 were purchased from DSMZ (Germany). Viable cell counts were determined using trypan blue staining. Cell lines were incubated at 37 °C with 5% CO2 with saturating humidity. according to DSMZ recommendations. MOLM-13 and THP-1 were maintained in 90% RPMI 1640 supplemented with 10% heat-inactivated FBS and 1% Streptomycin/Penicillin. OCI-AML6 was cultured in 80% alpha-MEM (containing ribonucleosides and deoxyribonucleosides), 20% heat-inactivated FBS, and 20 ng/mL GM-CSF and 1% Streptomycin/Penicillin. UCSD-AML1 was cultured in 80% RPMI 1640, 20% heat-inactivated FBS, and 20 ng/mL GM-CSF and 1% Streptomycin/Penicillin. All cells were tested for mycoplasma contamination using an Enzyme-based detection assay. Moreover, human cell line authentication was confirmed via short tandem repeat (STR) analysis at the Cytogenetics and Cell Authentication Core at the University of Texas MD Anderson Cancer Center within 6 months of conducting the experiments.

### Reagents and chemotherapeutics

For *in vitro* studies, VEN was purchased from Chemgood (Henrico, VA) in powder form, dissolved in DMSO to create 1–10 mM stock solutions, and stored at -80 °C. EBD-300 was supplied by Enzyme By Design (Chicago, Illinois) at a concentration of 400 IU/mL and stored at -80 °C.

For *in vivo* studies, EBD-300 was supplied by Enzyme By Design at 400 IU/mL and stored at -80 °C. It was diluted in the following sterile buffer solution: 25 mM Tris Buffer, pH 7.5; 200mM β-mercaptoethanol (BME) at the appropriate solution concentration. VEN powder was stored at 4 °C and then formulated fresh on each week of dosing at 10% DMSO, 30% PEG400, 60% Phosal PG50.

### Fish assay

A CytoCell Del(7q) Deletion probe specifically designed to detect chromosomal deletions at regions 7q22.1-q22.2 and 7q31.2 on chromosome 7, was used for this study. It utilizes a red-labeled probe (Texas Red) that covers a 396kb region at 7q22.1-q22.2 (including the telomeric end of the RELN gene), and a green-labeled probe (FITC) that covers a 203kb region at 7q31.2 (including the TES gene).

The signal count was performed by manually scoring the distinct fluorescent spots in each cell nucleus, up to 200 nuclei per sample to ensure statistical accuracy (as per MD Anderson’s CLIA approved standards).

### Cell proliferation assay

Cell lines were seeded into 96-well plates and treated with VEN, EBD, and their combination. Plates were incubated for 96 hours at 37 °C with 5% CO2 with saturating humidity. Subsequently, the plates were read using a Tecan Infinite plate reader (Tecan, Männedorf, Switzerland). Data were analyzed using GraphPad Prism Software (Graphpad, La Jolla, CA).

### Western blot analysis

Cells were lysed in protein lysis buffer (0.25 mol/L Tris-HCl, 2% sodium dodecyl sulfate (SDS), 4% β-mercaptoethanol, 10% glycerol, 0.02% bromophenol blue; 0.2 × 106 million cells in 10 μL). Equal amounts of protein samples were resolved by 10% or 12% SDS–polyacrylamide gel electrophoresis (PAGE),and then transferred to nitrocellulose membrane. Immunoblotting was performed with primary antibodies: *ASNS* (from Cell Signaling Technology, #20843) with 1:1000 dilution and β-actin 1:15,000 dilution. The samples were then incubated in secondary antibodies for 1 hour and washed 3 times in 1× phosphate-buffered saline with Tween 20 (PBST). Blots were scanned with Film Imaging System.

### Methylcellulose assays

Fresh primary AML peripheral blood samples were processed using the Ficoll density gradient centrifugation method as described previously. CD34+ cell enrichment was performed using the EasySep Human CD34 Positive Selection Kit from StemCell Technologies (#17856) following the manufacturer’s protocol. The enriched cells were resuspended in 2% IMDM medium at a concentration of 0.2 × 10^6^ cells/mL.

For the colony-forming unit (CFU) assays, 200,000 cells were plated in MethoCult H4435 medium from StemCell Technologies (#04435) in 12-well plates under four treatment conditions (VEH, VEN, EBD, VEN+EBD), with each condition tested in triplicate. Each well contained a total volume of 2000 μL methylcellulose medium, 100 μL cell suspension, and 100 μL drug suspension. The plates were incubated at 37 °C with 5% CO_2_ and 95% humidity for 14 days. Colonies were subsequently counted using an inverted microscope. For CFU scoring, aggregates of ≥100 cells were classified as colonies, while aggregates containing <100 cells were recorded as clusters. CFU counts were performed in a blinded manner with respect to treatment group.

### *In-vivo* study design

NOD/SCID mice were purchased from Jackson Laboratory (Sacramento, CA) and housed under standard conditions as approved by the 00002373-RN00 IACUC protocol at UT MD Anderson Cancer Center. Mice were randomized into treatment groups based on initial engraftment percentage, ensuring that each group had a balanced baseline engraftment range across cages. Randomization was performed by coded allocation to maintain concealment until treatment administration. Group sizes (n=8 mice per arm) were selected based on prior experience with AML PDX models, in which 3 mice were sacrificed on Day 24 to measure leukemia burden and other relevant markers, while the remaining 5 mice were followed for survival. This design provided sufficient power to detect treatment effects while minimizing animal use.

Humane endpoints were followed according to IACUC guidelines and included weight loss exceeding 20% of baseline, persistent recumbency, severe lethargy, inability to reach food, significant dehydration, dyspnea or abnormal breathing, and severe diarrhea. Mice meeting endpoint criteria were euthanized by CO_2_ inhalation followed by cervical dislocation. Supportive care, such as hydration gel and softened diet, was provided by veterinary technicians as needed based on daily observation. No analgesia was used in this study, as no surgical procedures were performed. All mice meeting endpoint criteria were excluded from the study and reported. All animal procedures were performed in accordance with MD Anderson IACUC protocols.

### *In-vivo* efficacy of EBD-300 in an AML PDX model conducted by champions oncology

Four-six-week-old female NOG mice were sub-lethally irradiated with 150 cGy whole-body irradiation (RS 2000 – X-ray Biological Irradiator, RadSources Technologies, Inc.) 4 hours prior to inoculation with AML cells from model CTG-2456. Irradiated mice were injected with 2 × 10^6^ viable human AML cells (0.2 mL in PBS) into the lateral tail vein. Starting two weeks after the cell inoculation, blood and bone marrow AML burden was analyzed by flow cytometry using a custom AML Flow panel at 1–3 time points prior to the estimated engraftment window based upon previously known engraftment kinetics. Once sufficient engraftment levels were observed in surrogate animals (%hCD45+ of live cells averages at ≥20%), the remaining pre-study animals were randomized to a vehicle control and EBD-300 groups with animals of similar body weight (n=10/group). EBD-300 was given iv at a dose of 750 IU/kg on Mondays and Wednesdays, and 1,500 IU/kg on Friday, for 28 days. Mice were monitored daily via clinical observations. Body weights were taken 3 times during the week post-implant and once weekly thereafter. At Day 28, as much whole blood as possible was collected via cardiac puncture from all groups. Blood was transferred to K2EDTA tubes and gently mixed by inversion (by hand) 8–10 times. After inversion, sample tubes were stored on wet ice until processed for flow analysis using the Standard AML panel. Bone marrow was collected for flow analysis by flushing the marrow through the tibia and femur from both legs with MACS media and pooled into collection tubes for each mouse. Samples were stored on wet ice until processed for flow cytometry.

### *In-vivo* efficacy of VEN, EBD-300 and VEN+EBD-300 in patient derived xenografts

Viable frozen PDX cells were thawed and washed with AlphaMEM supplemented with 20% fetal bovine serum (FBS). The following thawing medium was then prepared: 10 mL of warm AlphaMEM with 20% FBS, 200 μL of heparin, 200 μL of DNase, and 500 μL of MgSO_4_. The thawed PDX cells were incubated in this medium for 15 minutes at 37 °C. Following incubation, cells were centrifuged at 1500 rpm for 5 minutes, and the supernatant was discarded. The cells were then washed with 10 mL of PBS and filtered through a cap filter flow tube. After filtering, the cells were counted and prepared for injection. A total of 32 female NSG mice, aged 6–8 weeks, were used for the study. The mice were subjected to sublethal irradiation at 250 cGy 24 hours before cell injection. Each mouse was injected with 1 × 10^6^ PDX cells. After confirming robust engraftment, the mice were randomized into four groups of eight. Treatment began on the day of randomization with the following regimens: VEH control, Venetoclax (VEN) administered orally at 100 mg/kg, 5 days per week for two weeks, EBD-300 administered intraperitoneally at 750–1500 IU/kg, two to three times a week for three weeks, or a combination of VEN and EBD. Engraftment was monitored weekly by collecting peripheral blood and assessing human CD45 (hCD45) and murine CD45 (mCD45) levels. On Day 24, three mice from each group were sacrificed to measure leukemia burden in the spleen, bone marrow, and liver using hCD45 and mCD45 markers. The rest of the mice (5 mice per group) were monitored for survival.

### Toxicity study in CD-1 mice

Female CD-1 mice (n=5) were injected i.p. with EBD-300 at a dose of 2,000 IU/kg once a week for 4 doses. After the last dose was given, the mice were fasted for 18 hours and then sacrificed, and blood was collected for clinical chemistry analysis.

### Statistical analysis

For the viability and Annexin V/PI assays conducted in AML cell lines, data are presented as the standard error of the mean (SEM), with a significance level set at p<0.05. Dose-response curves were generated, and IC50 values were calculated by nonlinear regression analysis using a four-parameter logistic (4PL) model. Each value represents the result of 3 to 4 independent biological replicates. To compare the viability percentages among different treatment conditions, a one-way ANOVA followed by Tukey’s *post hoc* test was performed. Similarly, for the colony-forming unit (CFU) assays, a one-way ANOVA with Tukey’s *post hoc* test was used.

For the weight chart, which tracks the weights of each mouse in the different groups (VEH VEN, EBD-300, VEN+EBD-300) over the study timeline, a two-way ANOVA followed by Tukey’s *post hoc* test was utilized.

Lastly, mouse survival was analyzed using the Kaplan-Meier method, and comparisons between the four treatment groups were made. All statistical parameters, including sample sizes and p-values, are reported in the figures and their corresponding legends.

### Pharmacokinetics

Healthy wild-type CD-1 female mice (8–10 weeks) were injected intravenously with 4,000 IU/kg EBD-300 or intraperitoneally with either 2,500 IU/kg Spectrila (*Ec*A) or 7,500 IU/kg *Er*A. For the activity determinations, 50 µL of peripheral blood was collected in EDTA-coated tubes at regular intervals: 10 time points were collected over 120 hours for EBD-300; 6 time points were collected over 24 hours for *Ec*A; and 8 time points were collected over 10 hours for *Er*A. Samples were processed by centrifugation at 4 °C at 4,600 rpm (2000xg) for 10 min. The plasma was either tested immediately for asparaginase activity or flash-frozen in liquid nitrogen and stored at -80 °C.

## Data Availability

The original contributions presented in the study are included in the article/**Supplementary Material**, further inquiries can be directed to the corresponding author/s.

## Supplementary material

Supplementary Figure 1 The bacterial ASNases EcA and ErA show strong L-glutaminase activity whereas EBD-300 shows no glutaminase activity. L-GLNase activity in the presence of 5 mM glutamine was monitored by 1D H-NMR spectroscopy. EcA demonstrates strong L-glutaminase activity at a concentration of 2 µM (brown trace) and residual activity at 0.1 µM (orange trace); ErA demonstrates significant activity even at the low concentration of 0.1 µM (gray trace). In contrast, EBD-300 demonstrates a clear lack of GLNase activity at 0.1 µM (light blue trace) and 2 µM (blue trace).

Supplementary Figures 2–4 Full figures showing three biological replicates for each treatment condition in patients 1,2 and 3.

Supplementary Figure 5 (A) Survival analysis in PDX-1 showed a median survival of 52 days in the vehicle (VEH) group, 54 days in the Venetoclax (VEN) group, 49 days in the EBD-300 group, and 60 days in the VEN+EBD-300 combination group. However, the P-values were not statistically significant across all groups. (B) Additionally, the leukemia burden in the bone marrow, liver, and spleen in the VEH, VEN, EBD, and VEN+EBD-300 groups was not significant in PDX-1. The error bars shown represent the standard error of mean (SEM).

Supplementary Figure 6 (A) Groups treated with EBD, as well as the VEN+EBD-300 combination group, exhibited significantly more weight loss compared to the VEH and VEN groups in PDX-3. In PDX-2, survival analysis showed median survival times of 83 days in the VEH group, 91 days in the VEN group, 85 days in the EBD-300 group, and 64 days in the VEN+EBD-300 group. Although the difference between groups was not statistically significant, deaths in the EBD-300 and VEN+EBD-300 groups were likely due to significant weight loss. (B) Similar to (A), groups receiving EBD-300 and VEN+EBD-300 experienced significantly more weight loss in comparison to the VEH and VEN groups in PDX-3. The error bars shown represent the standard error of mean (SEM).

Supplementary Figure 7 Representative gating strategy illustrating the gating strategy for flow cytometry used to identify human cells within mouse spleen samples. Spleen cells were first gated on FSC-A vs SSC-A to exclude debris and select the main population of cells. Them, FSC-A vs FSC-H was used to exclude duplets and select for single cells. Live cells were then identified as DAPI-negative (FL-9) using DAPI as a viability stain. Finally, within the singlets population, cells were analyzed for the expression of human CD45 (FITC, FL-1) and mouse CD45 (APC, FL-6) to identify the human myeloid cells from mouse cells.

## References

[1] Stubbins RJ Francis A Kuchenbauer F Sanford D . Management of acute myeloid leukemia: A review for general practitioners in oncology. Curr Oncol (Toronto Ont.). (2022) 29:6245–595. doi: 10.3390/curroncol29090491, PMID: 36135060 PMC9498246

[2] Visani G Chiarucci M Paolasini S Loscocco F Isidori A . Treatment options for acute myeloid leukemia patients aged <60 years. Front Oncol. (2022) 12:897220. doi: 10.3389/fonc.2022.897220, PMID: 36276074 PMC9581198

[3] DiNardo CD Jonas BA Pullarkat V Thirman MJ Garcia JS Wei AH . Azacitidine and venetoclax in previously untreated acute myeloid leukemia. New Engl J Med. (2020) 383:617–29. doi: 10.1056/NEJMoa2012971, PMID: 32786187

[4] Bose P Gandhi V Konopleva M . Pathways and mechanisms of venetoclax resistance. Leukemia Lymphoma. (2017) 58:1–175. doi: 10.1080/10428194.2017.1283032, PMID: 28140720 PMC5478500

[5] Abbas HA Ayoub E Sun H Kanagal-Shamanna R Short NJ Issa G . Clinical and molecular profiling of AML patients with chromosome 7 or 7q deletions in the context of TP53 alterations and venetoclax treatment. Leukemia Lymphoma. (2022) 63:3105–16. doi: 10.1080/10428194.2022.2118533, PMID: 36089905 PMC9772202

[6] Asselin B Rizzari C . Asparaginase pharmacokinetics and implications of therapeutic drug monitoring. Leukemia Lymphoma. (2015) 56:2273–805. doi: 10.3109/10428194.2014.1003056, PMID: 25586605 PMC4732456

[7] Zhou R Liang T Li T Huang J Chen C . Possible mechanism of metabolic and drug resistance with L-asparaginase therapy in childhood leukaemia. Front Oncol. (2023) 13:1070069. doi: 10.3389/fonc.2023.1070069, PMID: 36816964 PMC9929349

[8] Riley DO Schlefman JM Von Eckstaedt V HCV Morris AL Keng MK El Chaer F . Pegaspargase in practice: minimizing toxicity, maximizing benefit. Curr Hematologic Malignancy Rep. (2021) 16:314–245. doi: 10.1007/s11899-021-00638-0, PMID: 33978914

[9] Van Trimpont M Peeters E De Visser Y Schalk AM Mondelaers V De Moerloose B . Novel insights on the use of L-asparaginase as an efficient and safe anti-cancer therapy. Cancers. (2022) 14:902. doi: 10.3390/cancers14040902, PMID: 35205650 PMC8870365

[10] Nguyeni HA Su Y Lavie A . Design and Characterization of *Erwinia chrysanthemi* L-Asparaginase Variants with Diminished L-Glutaminase Activity. Journal ofBiological Chemistry (2016) 291:17664–76. doi: 10.1074/jbc.M116.728485, PMID: 27354283 PMC5016162

[11] Schalk Amanda M. Alexandra Sartori Kelli Roberts Richard A. Rosser Seth J. Corey . Identification and Structural Analysis of an l-Asparaginase Enzyme from Guinea Pig with Putative Tumor Cell Killing Properties. J Biol Chem (2014) 289 (12):8359–67. doi: 10.1074/jbc.M113.531866, PMID: 25320094 PMC4246078

[12] Nguyen HA Su Y Zhang JY Antanasijevic A Caffrey M Schalk AM . A Novel L-Asparaginase with Low l-Glutaminase Coactivity Is Highly Efficacious against Both T- and B-Cell Acute Lymphoblastic Leukemias *In Vivo*. Cancer Res. (2018) 78:1549–60. doi: 10.1158/0008-5472.CAN-17-2106, PMID: 29343523 PMC5856643

[13] Van Trimpont M Schalk AM Hofkens K Peeters E T'Sas S Vandemeulebroecke K . A human-like glutaminase-free asparaginase is highly efficacious in ASNSlow leukemia and solid cancer mouse xenograft models. Cancer Lett. (2025) 611:217404. doi: 10.1016/j.canlet.2024.217404, PMID: 39709177 PMC12177098

[14] Bogenberger J Whatcott C Hansen N Delman D Shi CX Kim W . Combined venetoclax and alvocidib in acute myeloid leukemia. Oncotarget. (2017) 8:107206–22. doi: 10.18632/oncotarget.22284, PMID: 29291023 PMC5739808

[15] Zalma BA Ibrahim M Rodriguez-Polanco FC Bhavsar CT Rodriguez EM Cararo-Lopes E . Autophagy-related 7 (ATG7) regulates food intake and liver health during asparaginase exposure. J Biol Chem. (2025) 301:108171. doi: 10.1016/j.jbc.2025.108171, PMID: 39798881 PMC11850126

[16] Emadi A Kapadia B Bollino D Bhandary B Baer MR Niyongere S . Venetoclax and pegcrisantaspase for complex karyotype acute myeloid leukemia. Leukemia. (2021) 35:1907–24. doi: 10.1038/s41375-020-01080-6, PMID: 33199836 PMC10976320

[17] Place AE Stevenson KE Vrooman LM Harris MH Hunt SK O'Brien JE . Intravenous pegylated asparaginase versus intramuscular native Escherichia coli L-asparaginase in newly diagnosed childhood acute lymphoblastic leukaemia (DFCI 05-001): A randomised, open-label phase 3 trial. Lancet Oncol. (2015) 16:1677–90. doi: 10.1016/S1470-2045(15)00363-0, PMID: 26549586

[18] Gossai N Richards M Boman L Messinger Y Gernbacher S Perkins J . Symptomatic hyperammonemia with erwinia chrysanthemi-derived asparaginase in pediatric leukemia patients. J Pediatr Hematology/Oncology. (2018) 40:312–15. doi: 10.1097/MPH.0000000000001062, PMID: 29334534

[19] Bertuccio SN Serravalle S Astolfi A Lonetti A Indio V Leszi A . Identification of a cytogenetic and molecular subgroup of acute myeloid leukemias showing sensitivity to L-asparaginase. Oncotarget. (2017) 8:109915–23. doi: 10.18632/oncotarget.18565, PMID: 29299118 PMC5746353

[20] Tabe Y Konopleva M . Resistance to energy metabolism - targeted therapy of AML cells residual in the bone marrow microenvironment. Cancer Drug Resistance. (2023) 6:138–505. doi: 10.20517/cdr.2022.133, PMID: 37065866 PMC10099600

[21] Kreitz J Schönfeld C Seibert M Stolp V Alshamleh I Oellerich T . Metabolic plasticity of acute myeloid leukemia. Cells. (2019) 8:805. doi: 10.3390/cells8080805, PMID: 31370337 PMC6721808

[22] Pezeshki A Podder S Kamel R Corey SJ . Monosomy 7/del (7q) in inherited bone marrow failure syndromes: A systematic review. Pediatr Blood Cancer. (2017) 64. doi: 10.1002/pbc.26714, PMID: 28708320 PMC5937691

