# Correction: A mammalian, glutaminase-free asparaginase enhances venetoclax activity in preclinical AML models with chromosome 7 deletion

**DOI:** 10.3389/fonc.2026.1824040

**Published:** 2026-04-23

**Authors:** Dhabya Majid, Zhe Wang, Amanda M. Schalk, Bin Yuan, Qi Zhang Tatarata, Jessica L. Root, Araceli Isabella Garza, Mhd Yousuf Yassouf, Basant T. Gamal, Sammy Feri-Borgogno, Annie Hoai Nguyen, Marina Konopleva, Evelien Peeters, Ying Su, Patrick K Reville, Farhad Ravandi-Kashani, Arnon Lavie, Hussein A. Abbas

**Affiliations:** 1 Department of Leukemia, The University of Texas MD Anderson Cancer Center, Houston, TX, United States; 2 Department of Biochemistry and Molecular Genetics, College of Medicine, University of Illinois at Chicago, Chicago, IL, United States; 3 Enzyme by Design Inc., Chicago, IL, United States; 4 Department of Gynecologic Oncology and Reproductive Medicine, The University of Texas MD Anderson Cancer Center, Houston, TX, United States; 5 Rice University, Houston, TX, United States; 6 Department of Oncology, Montefiore Einstein, Bronx, NY, United States; 7 Department of Diagnostic Sciences, Ghent University, Ghent, Belgium; 8 Research Biologist, Biological Science Research and Development, Department of Veterans Affairs Medical Center, Chicago, IL, United States

**Keywords:** acute myeliod leukemia, AML, AML with deletion 7, combination treatment, leukemia, venetoclax, asparaginase

There were several errors in **Table 1** as originally published. The unit reported next to kcat was incorrect and should be “s^−1^”. In addition, the Michaelis–Menten constant was incorrectly written as km and should be corrected to “Km”, and the unit µM should be corrected to “mM”. For Km: the “m” is NOT subscript; “Km” correct, K_m_ incorrect.

The reference labels in column 1 were also incorrect: ErAref1 (10), EcAref2 (10), gpAref2 (11), and hAref2 (11) should be corrected to *Er*A (10), *Ec*A (10), *Gp*A (11), and *h*A (11). Furthermore, gpA should be capitalized as “GpA”. Lastly, in the Enzyme column, the enzyme names should be italicized “(*Er*A, *Ec*A, *Gp*A, *h*A)”.

The corrected version of **Table 1** appears below

**Table 1:** The table summarizes the catalytic efficiency k_cat_ and Michaelis-Menten constant Km for asparaginase and glutaminase activities of each L-asparaginase.

Enzyme	Asparaginase activity		Glutaminase activity				Mouse half-life
	k_cat_ (s^-1^)	Km (µM)	k_cat_ (s^-1^)	Km (mM)	k_obs_ at 1 mM (s^-1^)	glutaminase/asparaginase activities (%)	t_1/2_ (hr)
*Er*A (10)	207.5 ± 3.6	47.5 ± 3.5	26.84 ± 0.26	0.36 ± 0.02	19.22	13	2.3
*Ec*A (10)	44.4 ± 0.3	15.0 ± 0.5	0.89 ± 0.01	1.38 ± 0.09	0.36	2	3.5
EBD-300	70.0 ± 0.5	41.2 ± 1.2	0.01	ND	0.00	0	20.8
*Gp*A (11)	38.6 ± 1.4	57.7 ± 6.4	0.00	ND	0.00	0	ND
*h*A (11)	14.4 ± 0.4	2,960 ± 131^a^	ND	ND	ND	ND	ND

Additionally, the *in vivo* mouse half-life is included for each enzyme.

^a^ *hA* shows allosteric behavior, in line with Type I asparaginases (*EcA* is a Type II enzyme), and for these the Km is called [S]_0.5_ or K_0.5_.

ND, Not determined.

There was a mistake in the caption of **Table 1** as published. The caption stated is: “The table summarizes the catalytic efficiency (kcat/Km) and Michaelis–Menten constant (Km) for asparaginase and glutaminase activities of each L-asparaginase. Additionally, the *in vivo* mouse half-life is included for each enzyme”. The corrected caption of **Table 1** appears below:

“The table summarizes the catalytic efficiency k_cat_ and Michaelis-Menten constant Km for asparaginase and glutaminase activities of each L-asparaginase. Additionally, the *in vivo* mouse half-life is included for each enzyme”.

There was a mistake in the caption of **Figure 1** as published. The caption stated is:

”EBD-300 is human-like with very high sequence identity to the human homolog and has the required kinetic properties for achieving sustained asparagine depletion without perturbing glutamine. EBD-300 is highly human-like in protein sequence compared to the asparaginase domain from the guinea pig homolog truncated at residue 369 (GpA369) but especially compared to the clinical bacterial asparaginases from E. coli (EcA) and Erwinia (ErA) (A) surface representations of the tetramers where each protomer in the tetramer is shown in a different color (gray, green, blue or orange) and residues not identical to the human homolog are denoted in red. (B) Pie charts displaying % identity compared to the human enzyme where non-human is shown in red and identity to human is show in blue”. Please delete the first sentence in the **Figure 1** legend as shown below and add in word “protein sequence” as shown in the corrected caption below

The corrected caption of **Figure 1** should be:

“EBD-300 is highly human-like in protein sequence compared to the asparaginase domain from the guinea pig homolog truncated at residue 369 (*Gp*A369) but especially compared to the clinical bacterial asparaginases from E. coli (*Ec*A) and Erwinia (*Er*A) (**A**) surface representations of the tetramers where each protomer in the tetramer is shown in a different color (gray, green, blue or orange) and residues not identical to the human homolog are denoted in red. (**B**) Pie charts displaying % identity compared to the human enzyme where non-human is shown in red and identity to human is show in blue”.

There was a mistake in the caption of **Figure 4** as published. The caption stated is:

“EBD-300 monotherapy suppressed leukemic growth in a del7q AML model. Human AML cells with cytogenetics were implanted in NOG mice (n = 4) and after confirming engraftment were randomly assigned to a vehicle control group and an EBD-300 group, which was dosed at 750 IU/kg Mondays and Wednesdays and 1,500 IU/kg on Fridays for a total study duration of 28 days. On Day 28 mice were sacrificed, and bone marrow (A) and blood (B) were analyzed for the presence of hCD45. The animals experienced an initial weight loss 10–15% which then stabilized for the duration of the study (C). Statistical significance was determined using Student t-test (Prism 9.1.2). The error bars shown represent the standard error of mean (SEM)”.

The corrected caption of **Figure 4** should be:

“EBD-300 monotherapy suppressed leukemic growth in a del7q AML model. Human AML cells (Champions Oncology model CTG-2456) with cytogenetics 46, XX, del(7)(q22q36) [15] were implanted in NOG mice (n=10) and after confirming engraftment were randomly assigned to a vehicle control group (n=10) and an EBD-300 group (n=10), which was dosed at 750 IU/kg Mondays and Wednesdays and 1,500 IU/kg on Fridays for a total study duration of 28 days. On Day 28 mice were sacrificed, and bone marrow (A) and blood (B) were analyzed for the presence of hCD45. The animals experienced an initial weight loss 10-15% which then stabilized for the duration of the study (C). Statistical significance was determined using Student t-test (Prism 9.1.2). The error bars shown represent the standard error of mean (SEM)”.

There was an error in the format of the enzyme names EcA, ErA, GpA, and hA. The correct formatting should be: *Ec*A, *Er*A, *Gp*A, and *h*A.

The original version of this article has been updated.

