# Supplementary figures and images for: A mammalian, glutaminase-free asparaginase enhances venetoclax activity in preclinical AML models with chromosome 7 deletion
Source: Front Oncol. 2026 Feb 10;15:1606239. doi: 10.3389/fonc.2025.1606239 (PMC12930363; doi:10.3389/fonc.2025.1606239)

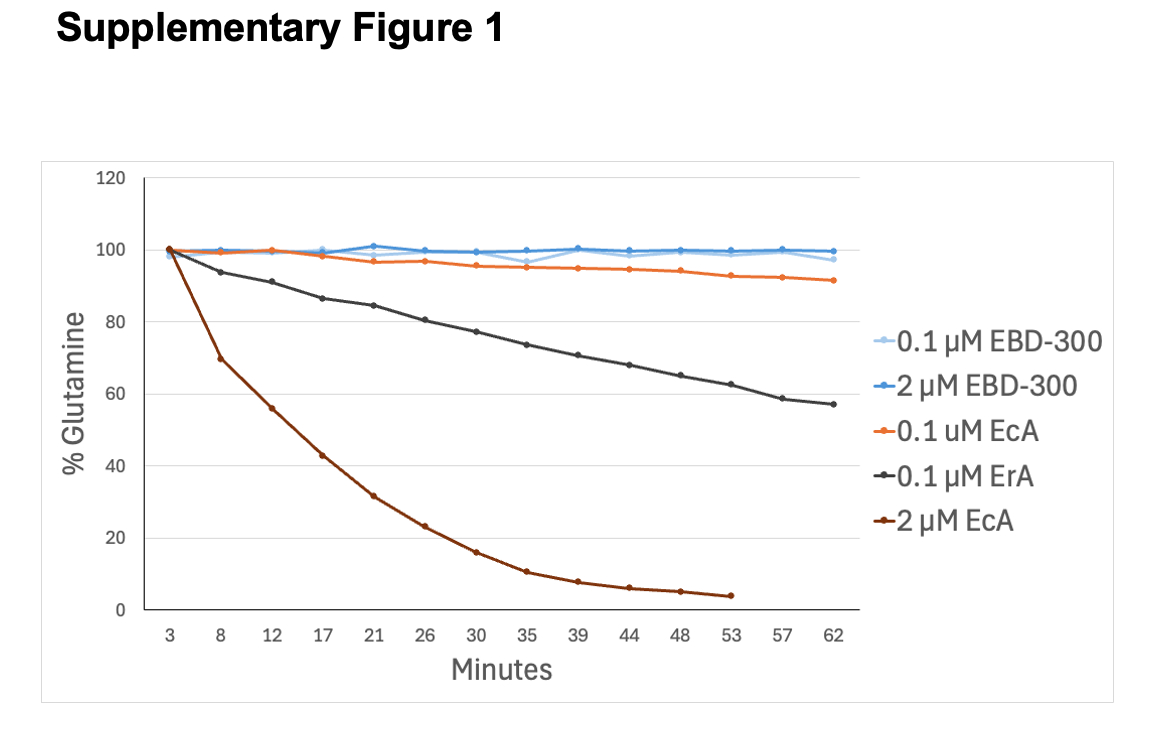

Supplement: Supplementary Figure 1 — The bacterial ASNases EcA and ErA show strong L-glutaminase activity whereas EBD-300 shows no glutaminase activity. L-GLNase activity in the presence of 5 mM glutamine was monitored by 1D H-NMR spectroscopy. EcA demonstrates strong L-glutaminase activity at a concentration of 2 µM (brown trace) and residual activity at 0.1 µM (orange trace); ErA demonstrates significant activity even at the low concentration of 0.1 µM (gray trace). In contrast, EBD-300 demonstrates a clear lack of GLNase activity at 0.1 µM (light blue trace) and 2 µM (blue trace). [file Image1.jpeg]

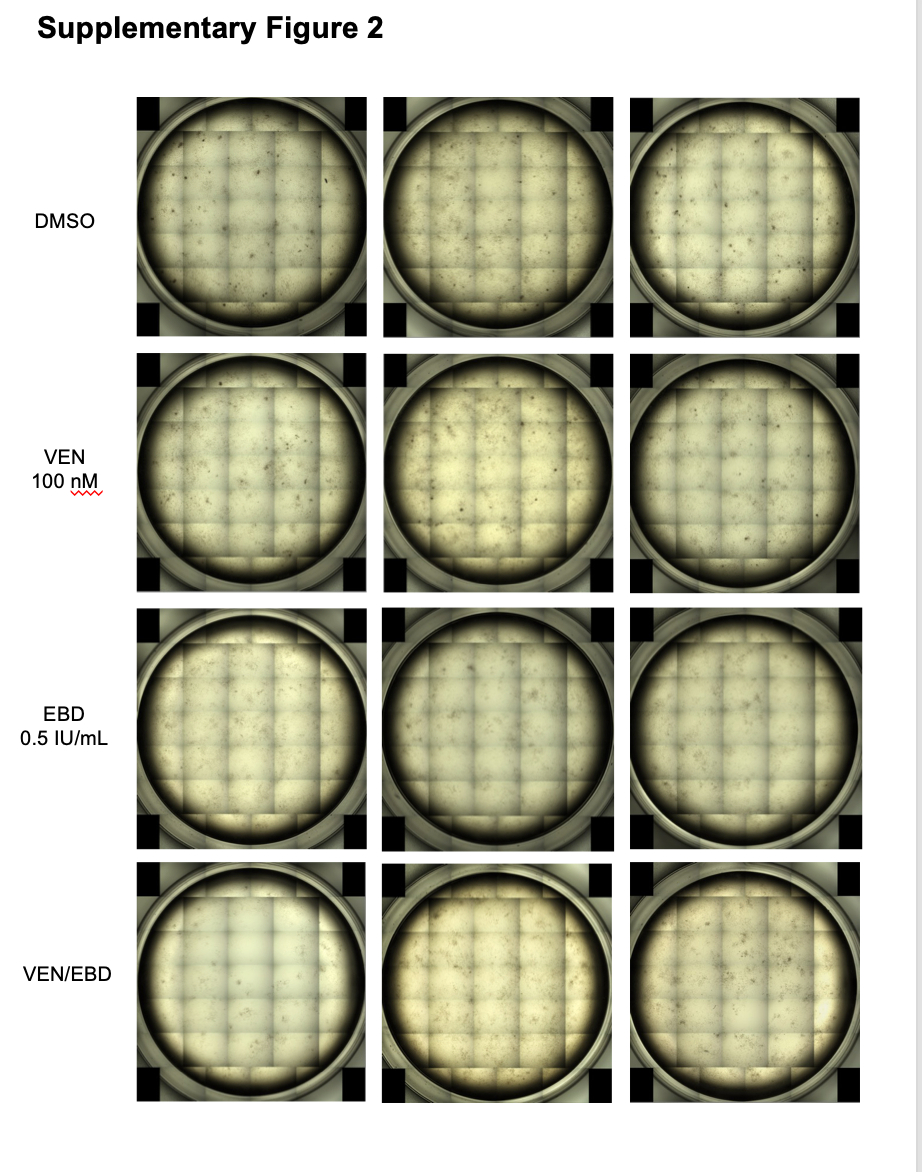

Supplement: Supplementary Figures 2–4 — Full figures showing three biological replicates for each treatment condition in patients 1,2 and 3. [file Image2.jpeg]

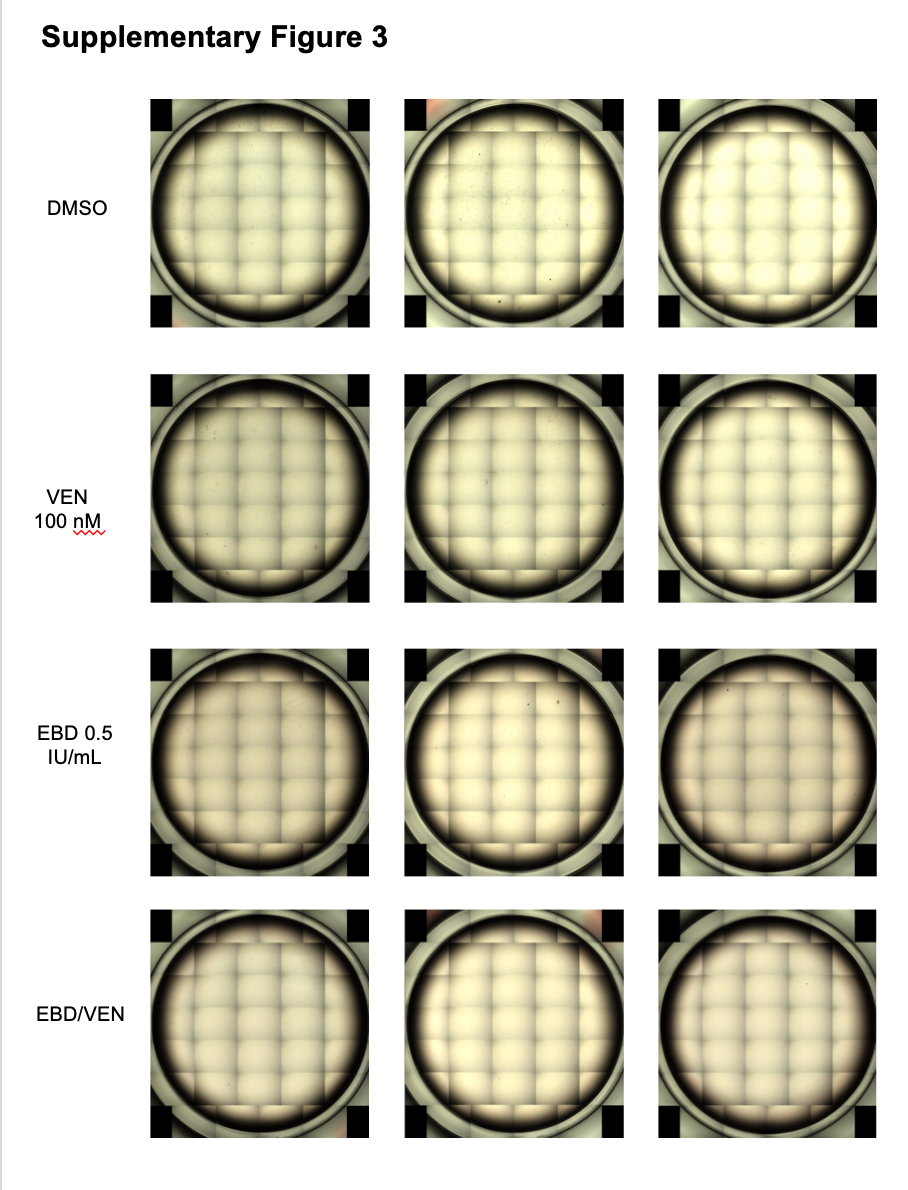

Supplement: Supplementary Figure 5 — (A) Survival analysis in PDX-1 showed a median survival of 52 days in the vehicle (VEH) group, 54 days in the Venetoclax (VEN) group, 49 days in the EBD-300 group, and 60 days in the VEN+EBD-300 combination group. However, the P-values were not statistically significant across all groups. (B) Additionally, the leukemia burden in the bone marrow, liver, and spleen in the VEH, VEN, EBD, and VEN+EBD-300 groups was not significant in PDX-1. The error bars shown represent the standard error of mean (SEM). [file Image3.jpeg]

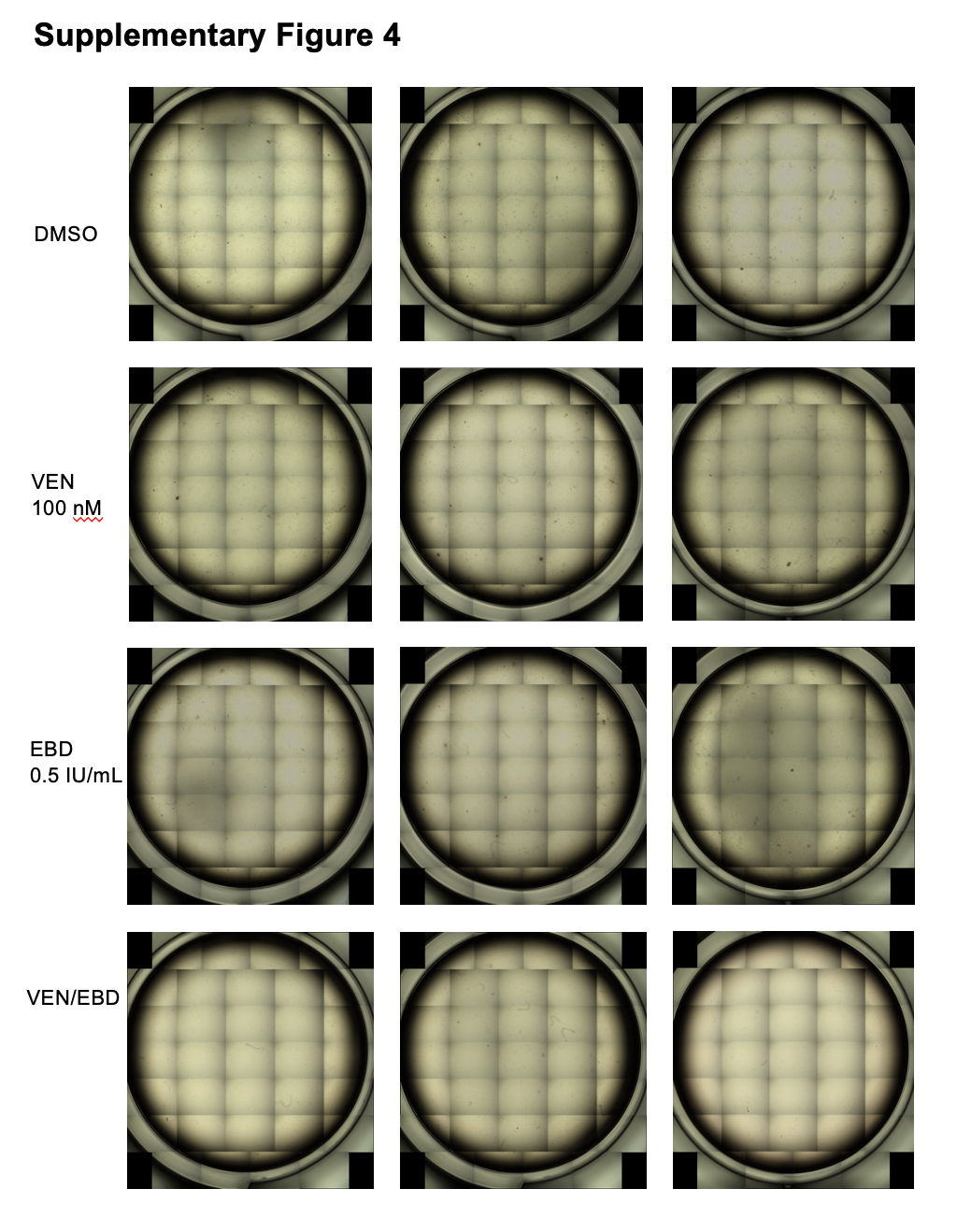

Supplement: Supplementary Figure 6 — (A) Groups treated with EBD, as well as the VEN+EBD-300 combination group, exhibited significantly more weight loss compared to the VEH and VEN groups in PDX-3. In PDX-2, survival analysis showed median survival times of 83 days in the VEH group, 91 days in the VEN group, 85 days in the EBD-300 group, and 64 days in the VEN+EBD-300 group. Although the difference between groups was not statistically significant, deaths in the EBD-300 and VEN+EBD-300 groups were likely due to significant weight loss. (B) Similar to (A), groups receiving EBD-300 and VEN+EBD-300 experienced significantly more weight loss in comparison to the VEH and VEN groups in PDX-3. The error bars shown represent the standard error of mean (SEM). [file Image4.jpeg]

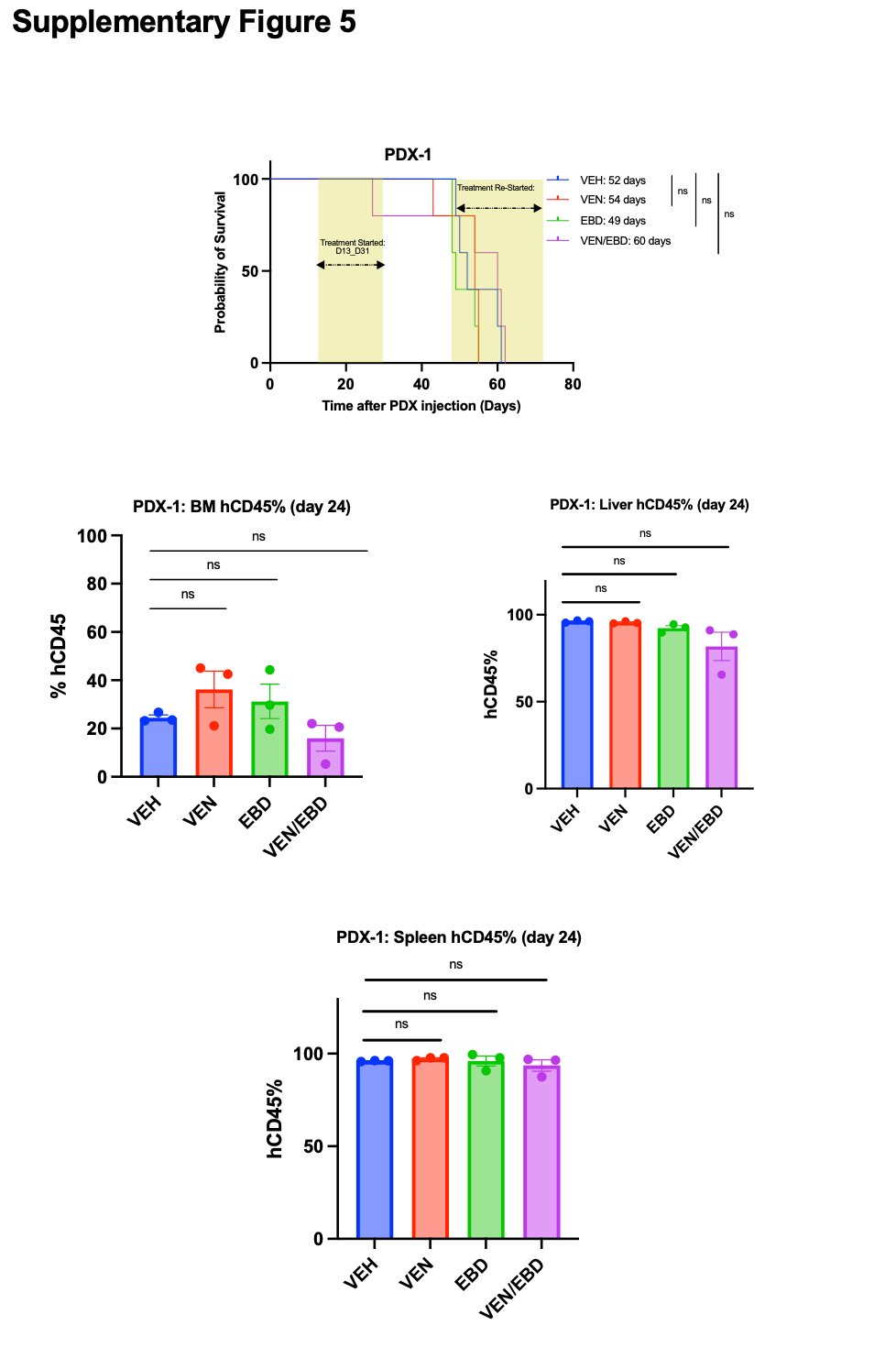

Supplement: Supplementary Figure 7 — Representative gating strategy illustrating the gating strategy for flow cytometry used to identify human cells within mouse spleen samples. Spleen cells were first gated on FSC-A vs SSC-A to exclude debris and select the main population of cells. Them, FSC-A vs FSC-H was used to exclude duplets and select for single cells. Live cells were then identified as DAPI-negative (FL-9) using DAPI as a viability stain. Finally, within the singlets population, cells were analyzed for the expression of human CD45 (FITC, FL-1) and mouse CD45 (APC, FL-6) to identify the human myeloid cells from mouse cells. [file Image5.jpeg]

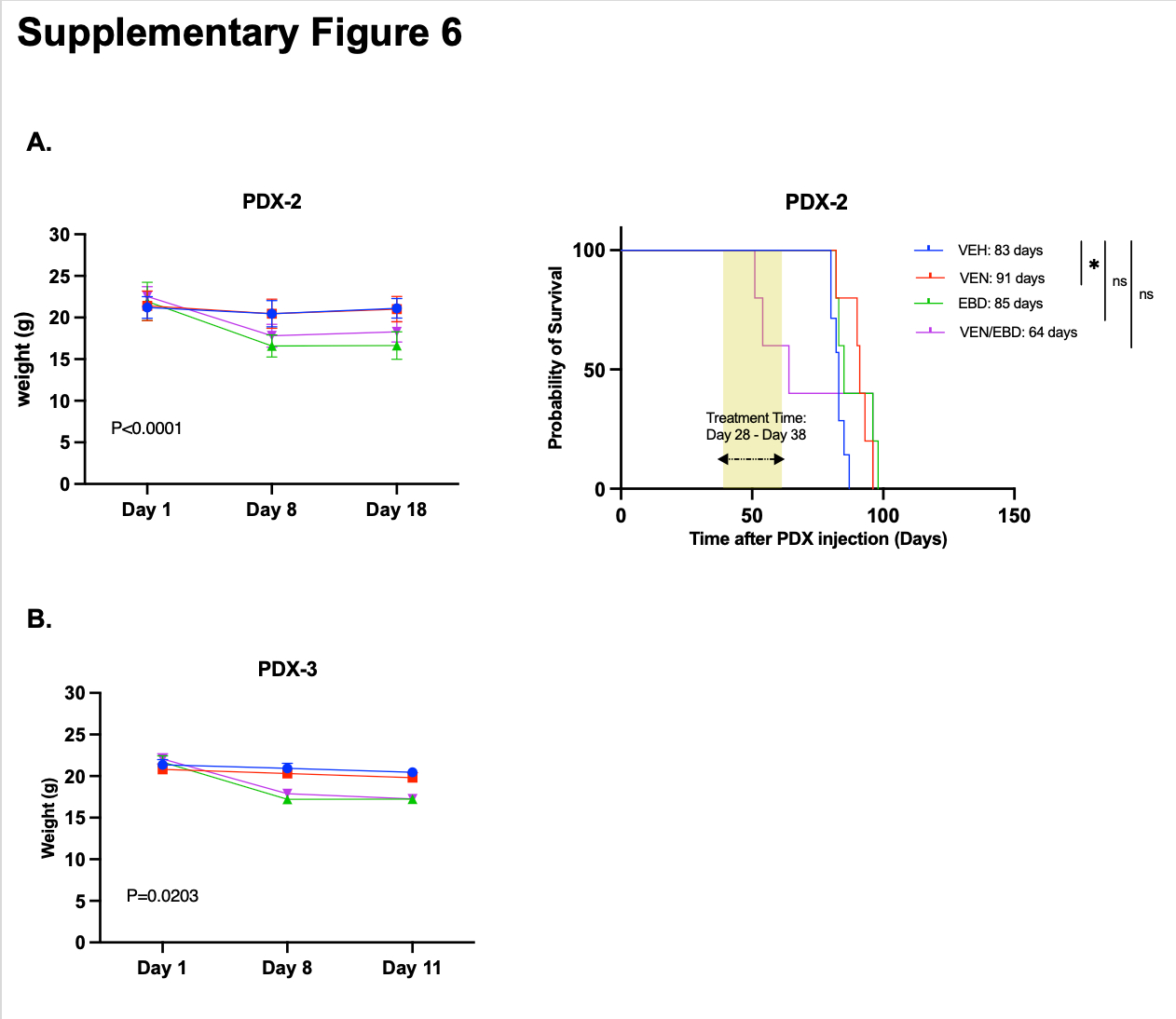

Supplement: Supplementary file 6 [file Image6.jpeg]

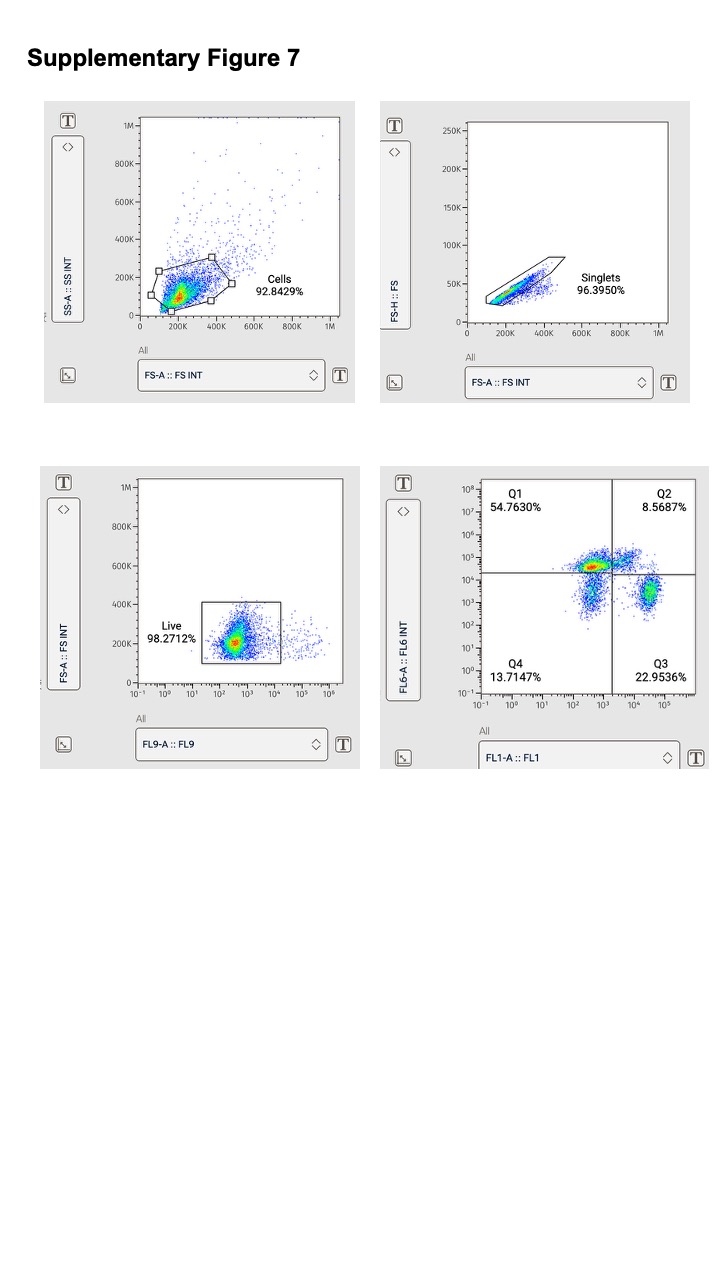

Supplement: Supplementary file 7 [file Image7.jpeg]
